# The diversity of resilience patterns of natural populations decreases closer to human activities

**DOI:** 10.1002/ecy.70557

**Published:** 2026-11-05

**Authors:** Thomas Merrien, Katrina J. Davis, Moreno Di Marco, Pol Capdevila, Roberto Salguero‐Gómez

**Affiliations:** ^1^ Department of Biology University of Oxford Oxford Oxfordshire UK; ^2^ Department of Biology and Biotechnologies “Charles Darwin” Sapienza University of Rome Rome Lazio Italy; ^3^ Departament de Biologia Evolutiva, Ecologia i Ciències Ambientals Universitat de Barcelona Barcelona Spain; ^4^ Institut de Recerca de la Biodiversitat (IRBio) Universitat de Barcelona Barcelona Spain

**Keywords:** demography, disturbances, ecology, environment, evolution, pressures, resilience

## Abstract

Quantifying the resilience of wild populations is essential to predict their viability in the Anthropocene. Many species have become threatened and even gone extinct because they have failed to cope with human disturbances. We use demographic data for 956 plant and animal populations to assess their responses to increasing levels of human activities. We show that in areas characterized by high human activities, there is a smaller diversity of resilience patterns compared to areas with lower human activities. Furthermore, in plants, populations closer to human‐influenced habitats display the ability to experience a lesser decrease in population size after disturbances than populations in less anthropized habitats. Our results suggest that human activities might have eroded the diversity of resilience patterns of wildlife populations, with key implications for the future resilience of wild populations worldwide.

## INTRODUCTION

Human activities, particularly since the industrial revolution, have fundamentally transformed Earth's life support systems (United Nations, 2019; Vitousek et al., 1997). These impacts include rapid increases in agricultural areas, now accounting for 37% of the land surface (Foley, 2005), and the growth of human‐made objects (buildings, infrastructure, products, etc.) beyond the cumulative mass of all living organisms worldwide (Elhacham et al., 2020). Recent estimates suggest that 59% of the Earth's terrestrial surface is now negatively impacted by human land use (Hurtt et al., 2020), leading to rapid decreases in species' ranges (Pacifici et al., 2020). More broadly, land‐use change is responsible for the loss of 5%–16% of vertebrate species compared to base levels from pristine habitats (Newbold, 2018), and has contributed to a 60% increase in vascular plant extinctions between 1900 and 2015 (di Marco et al., 2019). Ultimately, these fast and large‐scale changes in biodiversity affect the dynamics of the Earth's ecological systems (Yamaura et al., 2009) and their services (Dirzo et al., 2014). In fact, the homogenization of the environment due to land‐use modification is causing the loss of functional diversity worldwide (McKinney & Lockwood, 1999).

The impacts of human land use on the viability of wild populations can be directly quantified by assessing changes in their demography. Changes in the vital rates of survival, development, and reproduction within a wild population shape its ability to persist in a given location, ultimately determining a species' range of spatial distribution (Merow et al., 2014). As such, demographic approaches are powerful tools to examine population responses to human disturbances (Gamelon et al., 2011). We define disturbances here as events that cause temporary and localized shifts in those vital rates (Dornelas, 2010). Identifying the life history strategies that enable species to resist, recover from, and compensate for disturbances has become a promising research avenue to better understand the resilience of wild populations to human impacts (Capdevila et al., 2022). Recently, Capdevila et al. (2020) highlighted the important role of life history traits such as generation time in determining the resilience of wildlife populations. For instance, they showed that species with longer generation times take longer to recover from disturbances. Furthermore, human activities have important impacts on the resilience of plant and animal populations. For example, populations of black bears (*Ursus americanus*) or mule deer (*Odocoileus hemionus*) alter their activity patterns to become more active in periods where areas become less visited by humans (Lewis et al., 2021), and passerine populations host less diverse microbiomes in urban areas than in more pristine habitats (Teyssier et al., 2018). Although links have been established between life history traits and resilience on the one hand, and between human activities and species' activities and physiology on the other, it remains unclear whether and how human activities may alter life history strategies, with effects scaling up to population resilience. Our research proposes to investigate the nature and existence of this link.

Here, we examine how the resilience of 956 wildlife populations of 300 plant and 47 animal species worldwide correlates with human activities. To do so, we use life history traits and transient dynamics metrics that describe how populations respond to disturbances in the short term (Capdevila et al., 2020), hereby *demographic resilience* metrics. We test four hypotheses: (H1) The mean values of the three components of demographic resilience—resistance, speed of recovery, and compensation (i.e., ability to increase population size after a disturbance)—of natural populations will increase closer to habitats dominated by human activities, given that urban populations likely have confronted more disturbances than populations further from human activities, and might have adapted to them—or gone locally extinct (Sih et al., 2011); (H2) The three‐dimensional space defined by populations' resistance, speed of recovery, and compensation decreases closer to human disturbances, consistent with the expectation of erosion of functional diversity due to human activities (Carmona et al., 2021); (H3) The correlation between human activities and the resilience of a population depends on the species' capacity to move outside of the area of influence of human disturbance. Specifically, we expect the correlation between human activities and demographic resilience to be stronger in animals with limited mobility and all plants. Finally, we expect that (H4) Increases in human activities correlate with life history strategies that confer high demographic resilience. Because human activities often homogenize habitats (e.g., via urban development and agricultural monocultures [McKinney & Lockwood, 1999]), these activities might lead to a convergence in demographic responses to disturbances near human settlements. Also, because life history theory predicts that body size structures demographic rates and transient dynamics (Gaillard et al., 1989; Stearns, 1977), and because this trait correlates with resilience metrics (Capdevila et al., 2022), we accounted for them as potential covariates rather than as focal predictors, a common pre‐step in comparative demographic studies (Stott et al., 2024). We find that the diversity of resilience patterns is negatively correlated with the level of human activity.

## MATERIALS AND METHODS

### Human activity data

To quantify human activities, we used the Human Footprint (HFP; Venter et al., 2016), which correlates strongly with species extinction risk (di Marco et al., 2018; Ramírez‐Delgado et al., 2022). The HFP is composed of eight layers that cover all terrestrial surfaces of the globe, excluding Antarctic land areas, at a 1 km^2^ resolution. The HFP assigns a score out of 50 based on multiple human activities on the environment at each pixel (where a pixel is 1 km × 1 km). These eight layers of activity include human population density, roads, navigable waterways, railways, nighttime lights, built environment, pasture lands, and croplands. Two sets of maps are available in the HFP, for 1993 and 2009. These two points in time allow for a more comprehensive fit between the demographic data used here and the human activities at the time of the record. Specifically, for each demographic record, we selected the HFP record (1993 or 2009) that was closest to the period of time when the demographic data were collected in the field. Hence, if demographic data were collected in 2012 for a given species, the 2009 HFP snapshot would be more accurate to describe their human environment. Similarly, if demographic data were collected in 1987 for another species, the 1993 snapshot would better describe their human environment. To assess the robustness of our results to potential temporal mismatch between demographic data and the HFP, we repeated all phylogenetically and spatially corrected analyses using only demographic studies conducted within 1993–2009 (*n* = 226 populations from 84 plant species; 65 populations from 33 animal species). This brute‐force sensitivity analysis ensures direct temporal alignment between human pressure estimates and demographic observations.

We used multivariate analyses to assign a human‐pressure score to each of the wild populations we assessed. We first performed a principal components analysis (PCA) on all eight HFP layers. To do so, we rescaled the HFP map at a 10 km × 10 km resolution, subsampling just one pixel in each grid cell. Next, we extracted 60,000 random data points from the 10 km^2^ map and used that subset of data to run the PCA analysis. Because the HFP data were linked to the 1993 or the 2009 HFP version, we compared the PCA outputs coming only from the 1993 map, from the 2009 map, or from a mix of both—no detectable differences were found. Thus, we used the subset of 60,000 random but unique points distributed around the map and the two versions of the HFP. This number was chosen to be sufficiently high to reflect the diversity of locations around the world and be dimensioned to guarantee a reasonable calculation time for the PCA. We retained two dominant axes of variation of human activities following a sequence of criteria. First, using Kaiser's criterion (Legendre & Legendre, 2012), we retained the first three principal component axes because their associated eigenvalues >1 (Appendix S1: Table S1). Moreover, as PC3 had no direct biological interpretation and only explained 13%, we here focus on PC1 and PC2, which together explain >50% of the variance.

### Provenance and selection of demographic data

To obtain population‐structured data of multiple species, we used the COMPADRE Plant Matrix Database v.5.0.0 (Salguero‐Gómez et al., 2015) and the COMADRE Animal Matrix Database v.3.0.0 (Salguero‐Gómez, Jones, Archer, et al., 2016). These databases contain 9121 matrix population models (MPMs) of 760 plant species (“list_plant_species.pdf” in Merrien et al., 2026b, https://doi.org/10.5281/zenodo.22649459) and 2277 MPMs of 446 animal species, respectively (“list_animal_species.pdf” in Merrien et al., 2026b, https://doi.org/10.5281/zenodo.22649459). MPMs are discrete‐time structured population models in which the vital rates (e.g., survival, reproduction) are explicitly examined by classifying individuals into discrete stages (Caswell, 2000). We then calculated transient (short‐term) dynamics metrics that relate to key demographic resilience quantities (Capdevila et al., 2020), vital rates (Caswell, 2000), and life history traits (Salguero‐Gómez, Jones, Jongejans, et al., 2016). These calculations used the mean (across time) MPM under unmanipulated conditions for a given population to summarize the dynamics of each of our 956 populations across their examined period.

To ensure fair comparisons across populations within and across different species, we imposed a series of selection criteria on the 11,398 MPMs available in COMPADRE and COMADRE. We first removed species whose MPMs did not describe the full life cycle of the species. Next, we retained only terrestrial populations, since the HFP database focuses on terrestrial territories only (Venter et al., 2016), and populations where we could access the full HFP index, as in some cases layers were not quantified. We also discarded MPMs whose population growth rate (λ) ≥3, because such growth rates are rarely observed in natural populations given density‐dependent regulation and demographic constraints (Hernandez et al., 2025). We removed MPMs from populations that were subject to experimental manipulations (e.g., prescribed burning regimes) to link short‐term population dynamics under “control” conditions to human activities. Finally, we only kept MPMs that are ergodic, primitive, and irreducible (Frobenius et al., 1912; Perron, 1907) using the R package popdemo (Stott et al., 2012), so we could calculate key demographic metrics like population growth rate and transient metrics. Species with a single population were not discarded from the study, as our primary objective here is to examine the ecological patterns and correlates of demographic resilience, rather than to elucidate plausible evolutionary mechanisms (but see *Discussion*) (Appendix S1: Table S1 and “list_plant_species.pdf” in Merrien et al., 2026b at https://doi.org/10.5281/zenodo.22649459).

### Demographic resilience framework

To estimate the demographic resilience of populations across different species, we used Capdevila et al.'s (2020) framework based on metrics of transient dynamics (Stott et al., 2011). This framework is based on the analyses of the changes in population size and structure after a disturbance. When a disturbance is strong enough to force a population out of its demographic stable structure, a short‐term (transient) dynamic will emerge before converging to demographic stability again. The amplitude and duration of this short‐term transition can be quantified using transient dynamics. These transient metrics allow us to estimate the transient envelope, that is, the increase or drop in growth compared to the stable state of the population, as well as the time and speed required to reach stability again. We used these transient metrics to quantify each population's resistance, speed of recovery, and compensation when affected by pulse (i.e., one‐off) disturbances that may push the population away from its stationary equilibrium. Resistance here is quantified as the degree to which a population can retain its size and structure when disturbed (Capdevila et al., 2020). Speed of recovery describes the rate at which a population recovers its stable population growth rate. Finally, compensation describes the potential increase in population size caused by the pulse disturbance, with high compensation values corresponding to a population with the inherent potential for larger increases in population size post‐disturbance. We calculated resistance as the first‐step attenuation, ${\underline{\rho }}_{1}=\text{minCS}\left(\hat{A}\right)$ (Stott et al., 2011). This metric measures the population attenuation bound after a disturbance (Capdevila et al., 2020). Speed of recovery was calculated as the damping ratio, $\rho =\lambda_{1}/\|\lambda_{2}\|$. This dimensionless metric quantifies the speed of recovery to convergence towards stable growth (Capdevila et al., 2020). Finally, compensation was calculated as the maximum amplification ${\underline{\rho }}_{\max}=\max_{t>0}\left({\left\|{\hat{A}}^{t}\right\|}_{1}\right)$. Here, ***Â*** is the normalized ***A*** MPM, which involves scaling each element *a*_*i,j*_ of ***A*** by λ_*1*_; minCS is the minimum column sum of a matrix, λ_*1*_ is the dominant eigenvalue, and λ_*2*_ is the subdominant eigenvalue. Maximum amplification quantifies the amplification of population size after a pulse disturbance, that is, a compensation for post‐disturbance reductions in population size (Capdevila et al., 2020). To adhere to assumptions of normality in the residuals of the models we employ next, we logged the metrics of speed of recovery and compensation.

To assess whether the observed resilience hypervolume reflects nonrandom structuring across human pressure gradients (PC1 and PC2 in Figure 1), we compared the empirical convex hull volume to volumes generated under four null expectations, following Díaz et al. (2016). These null models test whether the observed joint distribution of resistance, recovery speed, and compensation differs from what would be expected if resilience components were distributed independently of human pressure or without multivariate constraint. The four distributions are: (1) Random distribution: trait values were randomly reassigned among populations, preserving marginal distributions but removing multivariate structure; (2) Uniform distribution: trait values were drawn uniformly between the observed minimum and maximum values, representing no clustering within the observed bounds; (3) Normal distribution: trait values were simulated from normal distributions matching observed means and variances; and (4) Gamma distribution: trait values were simulated from gamma distributions to account for potential skewness in resilience metrics. By comparing observed and simulated hypervolumes, we test whether the resilience space occupied by real populations is more constrained than expected under these distributional assumptions.

**FIGURE 1:**
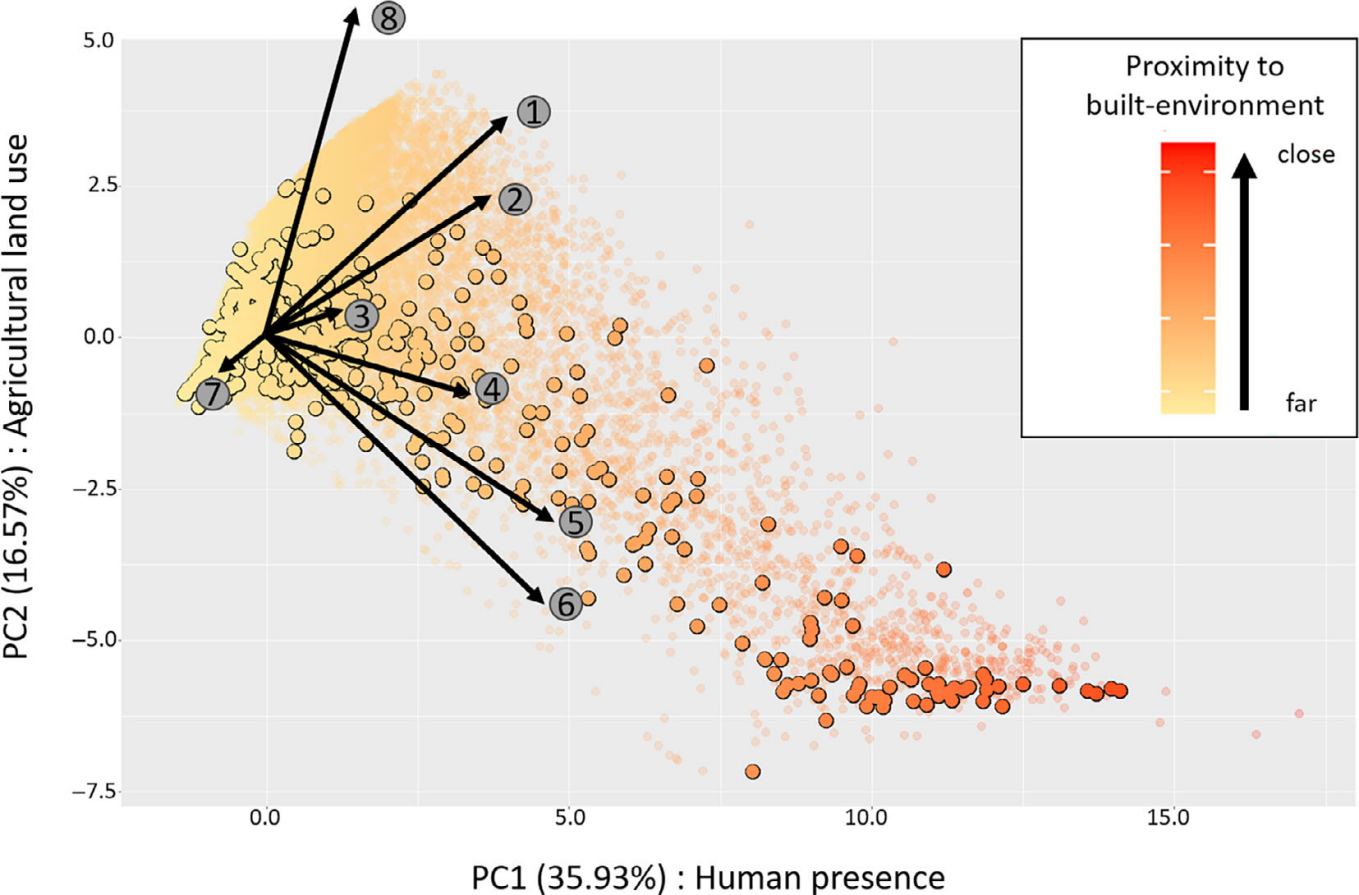
Principal components analysis (PCA) biplot of terrestrial habitats worldwide, derived from the Human Footprint database. The PCA shows two dominant axes of variation: PC1 corresponds to variation in human presence, and it is positively correlated with (1) human population density, (2) roads, (3) navigable waterways, (4) railways, (5) light pollution, (6) built environment, and it explains 35.93% of variation. PC2 differentiates locations according to land use, and it represents a trade‐off between (7) extensive pastures and (8) intense croplands, and it explains 16.57% of variation. Each point corresponds to a terrestrial unit of 10 km^2^ and is color‐coded according to its distance to a human‐built environment, here defined as human‐produced areas that provide the setting for human activity (delimited using satellite imagery). Points with a black circle correspond to Earth locations that contain at least one of our examined 956 wild populations.

To model the gamma distribution, we used the following parameters:

$$k=\frac{{\underline{x}}^{2}}{S^{2}};\;\theta =\frac{S^{2}}{\underline{x}}$$

with *k* being the shape and θ the scale of the gamma distribution, $\underline{x}$ the mean of the observed data, and *S* ^2^ the variance of the observed data.

To compute the smallest convex hull that encloses our data, we used the *geometry* R package (Roussel et al., 2023) and the *convhulln* function. Next, we simulated 1000 convex hulls following our four null hypotheses (1–4, above) and compared the volume occupied by the simulated hull to the volume occupied by our wild populations. We also used the *intersectn* function in *Base* R to quantify the percentage of overlap between the simulated and observed data.

To evaluate the functional diversity in transient metrics of our wild populations, we used the R package *BAT* (Mammola & Cardoso, 2020). We discretized PCA scores along PC1 and PC2 to facilitate kernel‐based estimation of functional diversity within comparable pressure classes. Bins were constructed to balance resolution across the gradient while ensuring adequate sample size (>30 populations per bin). We divided the human presence axis (PC1 in Figure 1) into five bins of approximately equal length ([<−2, 2.5[, [2.5, 6.5[, [6.5, 10.5[, [10.5, 14.5[, and [14.5, >18]) and attributed each population to the human presence level it is associated with. The number of bins was set to find a balance between there being enough data in each bin to carry out robust statistical analyses (>30 data points/bin) and having enough bins to properly describe the distribution of the data and the progression of the expected changes. We also carried out the same approach with the agricultural land use axis (PC2 in Figure 1), using three bins of approximately equal lengths given the distribution of our data ([<−7, −3.5[, [−3.5, 0.5[, and [0.5, >4]). In this case, three bins were enough to capture the distribution of the data. We then used the kernel approach (Mammola & Cardoso, 2020) to evaluate the richness (α‐diversity), the evenness, and the dispersion of our data. This step was implemented to assess more precisely the distribution of resilience metrics in highly pressured habitats and in less pressured habitats, thus allowing us to confirm the analyses we implemented with the convex hull. To verify robustness, we re‐ran kernel analyses using alternative bin numbers (e.g., five bins for both axes), which yielded qualitatively similar patterns (not shown). As each bin along both segmented axes did not have the same number of populations, we also ran 10 rarefaction analyses and built for each bin 10 kernels from 32 and 161 randomly selected populations for the human presence and agricultural land use axes, respectively. For each of the rarefaction analyses, we again estimated richness, dispersion, and evenness of the kernel. As the normality assumption was not achieved within the bins, we ran Kruskal–Wallis tests to test whether the level of human presence or agricultural land use is correlated with the functional diversity of resilience metrics. To test which segments were different from our less disturbed segment ([14.5, >18] in PC1, [0.5, >4] in PC2), we carried out pairwise comparisons of the different segments via Mann–Whitney‐Wilcoxon *U* tests.

### Provenance of additional traits

We used publicly available databases to collect additional traits needed to test our hypotheses. Height for each plant species was gathered through the TRY plant trait database (Kattge et al., 2020) and through various available sources detailed in “plant_height_sources.pdf” at https://doi.org/10.5281/zenodo.22649459 (Merrien et al., 2026b). For animals, average body mass data were collected from the Phylacine (Faurby et al., 2018) and Amniote life history databases (Myhrvold et al., 2015). Additional information was gathered from the MOSAIC database (Bernard et al., 2023).

### Phylogenetic information

To quantify and explicitly consider the effect of potential phylogenetic covariation in traits in our analyses, we constructed two phylogenetic trees resolved down to the population level for plants and animals. We used Timetree (Kumar et al., 2017) to create an animal phylogeny and the package V.phylomaker (Jin & Qian, 2019) to create a plant phylogeny. To accommodate multiple populations per species, we edited our phylogenies at the population level instead of the species level (see Online Appendix at https://doi.org/10.5281/zenodo.22649459; Merrien et al., 2026b).

### Demographic trait regression

To study the potential correlations between human activities and species' demographic resilience components, we used phylogenetically and spatially corrected models. Human activities were described according to the two dominant axes of variation in human activities estimated through the PCA (Figure 1). The correlations between human activities and demographic traits were based on the study of the residuals after removing the covariates known to influence demographic rates and transient dynamics: animal size/plant height (Capdevila et al., 2022) and spatial autocorrelation (Jongejans & de Kroon, 2005). We undertook this critical step to account for autocorrelations due to location and ancestry (Felsenstein, 1985; Legendre, 1993; Soler et al., 2013). We incorporated a correction for body mass and plant height (size, hereafter) (Gaillard et al., 2005; Oli & Dobson, 2003; Salguero‐Gómez, Jones, Jongejans, et al., 2016) in our study when linearity was assessed in the relationship between the trait of interest and size. Next, we assessed the effect of spatial autocorrelation on our models by calculating Moran's index (Moran, 1950) associated with the different traits or with the residuals of the regression of the trait and size when a size effect was detected. If spatial autocorrelation was found, we incorporated a correction for the location in the model (Zhang et al., 2005). To do so, we used the *tensor* function of generalized additive models (GAMs) with longitude and latitude (Wood & Augustin, 2002; Zuur, 2012) using R package *mgcv* version 1.8‐28 (Wood, 2011) (Equation 1).

Once the residuals of the above models were obtained, the second step was to carry out a phylogenetically corrected generalized linear mixed model of these residuals of the traits corrected (or not) for size and spatial autocorrelation on the HFP PCA main dimensions, and that considered the phylogenetic relationships among the species' populations (Equation 2).

(1) $$Y=\alpha_{1}+\beta_{1}.\text{size}+s\left(\text{Longitude},\text{Latitude}\right)+{ℇ}_{1},\;{ℇ}_{1}\sim Ν\left(0,\sigma_{1}^{2}\right),$$

(2) $$\mathrm{Res}\left(\left(\mathrm{Eq}.\;1\right)\right)=\alpha_{2}+\beta_{2}.\mathrm{PC}1+\alpha_{1}+\beta_{3}.\mathrm{PC}2+{ℇ}_{2},{ℇ}_{2}\sim Ν\left(0,\sigma_{2}^{2}\;V+\sigma_{2}^{2}\;I\right).$$

where *Y* is the trait of interest, PC1 and PC2 are the first two axes of the HFP PCA (Figure 1), Res(Eq. 1) are the residuals of the gam models in Equation (1), and *V* is the covariance matrix derived from the phylogeny and $\sigma_{2}^{2}\;I$ defines the non‐phylogenetic residual variance. We then used the *phyr* package (Li et al., 2020) and the function *pglmm* to run the phylogenetically corrected models.

All the analyses were performed in R version 4.0.1 (Team, R. C, 2021).

## RESULTS

### More resilient species in highly disturbed habitats?

Our HFB PCA analyses show that two axes of human activity are biologically meaningful: human presence and agricultural land use. Together, these two axes explain >52% of the variance in human activities (Figure 1). Higher‐order principal components explained additional variance but lacked clear biological interpretability (Appendix S1: Table S1). The first axis, PC1 (“Human presence axis” hereafter), absorbs 35.86% of the variance and separates areas with high human population density, high degree of built environment, and high light pollution (Figure 1, right) versus areas with low human presence (Figure 1, left). The second axis, PC2 (“Agricultural land use axis” hereafter), absorbs 16.63% of the variance and separates intensive agricultural croplands (Figure 1, top) from extensive pastures (bottom).

Our analyses reveal that plant populations closer to human settlements (right of PC1 in Figure 1) have higher resistance compared to plant populations in less human‐disturbed habitats (*Z* = 2.038, *p* = 0.042; Figure 2A; Appendix S1: Table S2). The compensation and speed of recovery of plants do not correlate with human activities (PC1: *Z* = −1.741, *p* = 0.082; *Z* = 1.945, *p* = 0.052, and PC2: *Z* = −0.665, *p* = 0.506; *Z* = 0.779, *p* = 0.436, respectively). In the case of animals, no metric of the demographic resilience correlates with either the human presence axis (PC1 in Figure 1) or the agricultural land use axis (PC2 in Figures 1 and 2). Our correlation tests toward PC1 and PC2 remain largely insensitive to the well‐established relationship between demographic resilience metrics and generation time (Gamelon et al., 2014; Appendix S1: Table S3), but did exhibit differential patterns when matrix dimensionality (Stott et al., 2011) was explicitly considered (Appendix S1: Table S2). As such, in what follows, we show the results with matrix dimensionality as a covariate in our analyses.

**FIGURE 2:**
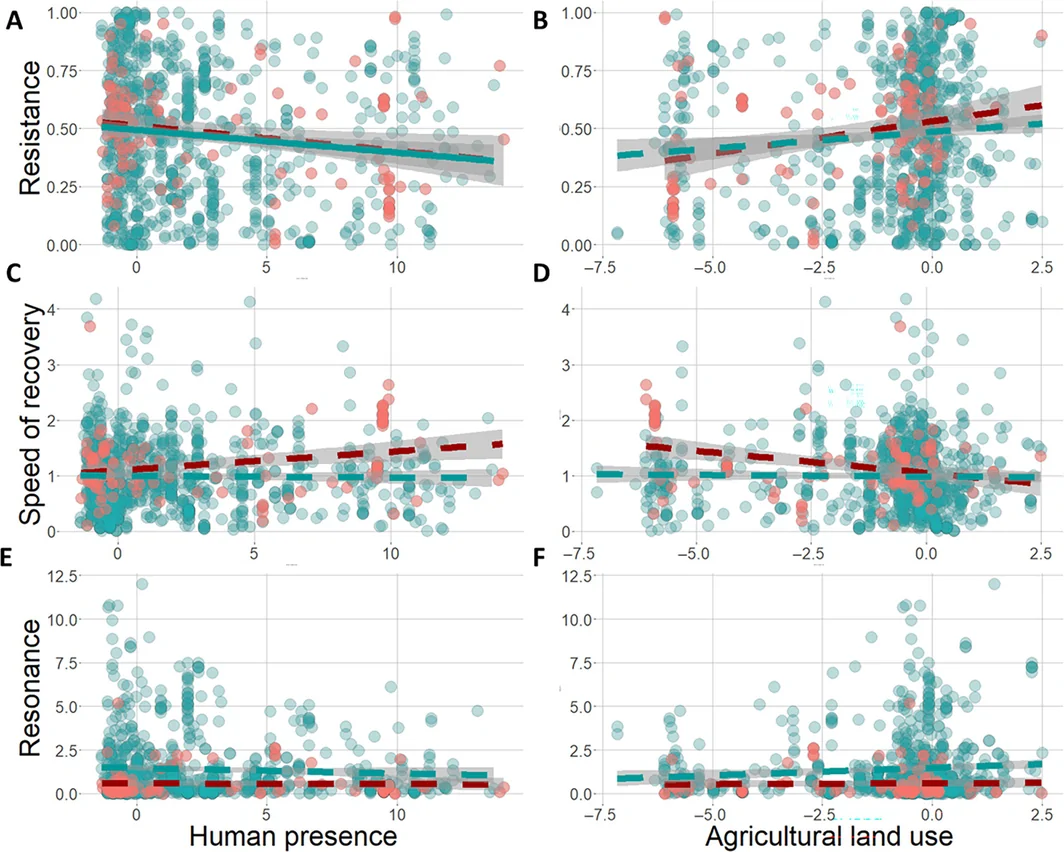
The three components of demographic resilience—resistance, recovery, and compensation—correlate with human activities in different ways for plants and animals. Correlation of three key demographic resilience components of our studied 956 wild populations along the continua of human presence (A, C, E) and agricultural land use (B, D, F) (see Figure 1). The *y*‐axes show the three components of the demographic resilience after accounting for the effect of the spatial autocorrelation in the data. The linear fits describe the phylogenetically and spatially corrected correlation between the predicted values from the model and the two selected axes of human activities, with blue for plants and red for animals. Not statistically significant (*p* > 0.05) correlations are shown as dashed lines; solid lines when significant (*p* < 0.05).

### The resilience hypervolume: Human activities constrain the spectrum of resilience patterns

The repertoire of resilience patterns defined by the three components of demographic resilience decreases sharply in more densely human‐populated areas (Figure 3) and more intensively farmed areas. The observed volume of resilience patterns along the human presence axis is different from that expected under normal, gamma, or uniform distributions (Table 1). Indeed, the volumes occupied by the resistance, speed of recovery, and compensation of our 956 wild populations as a function of their proximity to human settlements under an assumption of uniform, gamma, or normal distributions are always larger than our observed data (Table 1). Furthermore, none of the simulated distributions fully overlap our field data (Table 1). Especially for the normal distribution, the intersection between the simulated and observed data leaves out more than 30% of the observed data. The distribution of the three components of demographic resilience along the agricultural land use axis behaves similarly to the distribution along the human presence axis.

**FIGURE 3:**
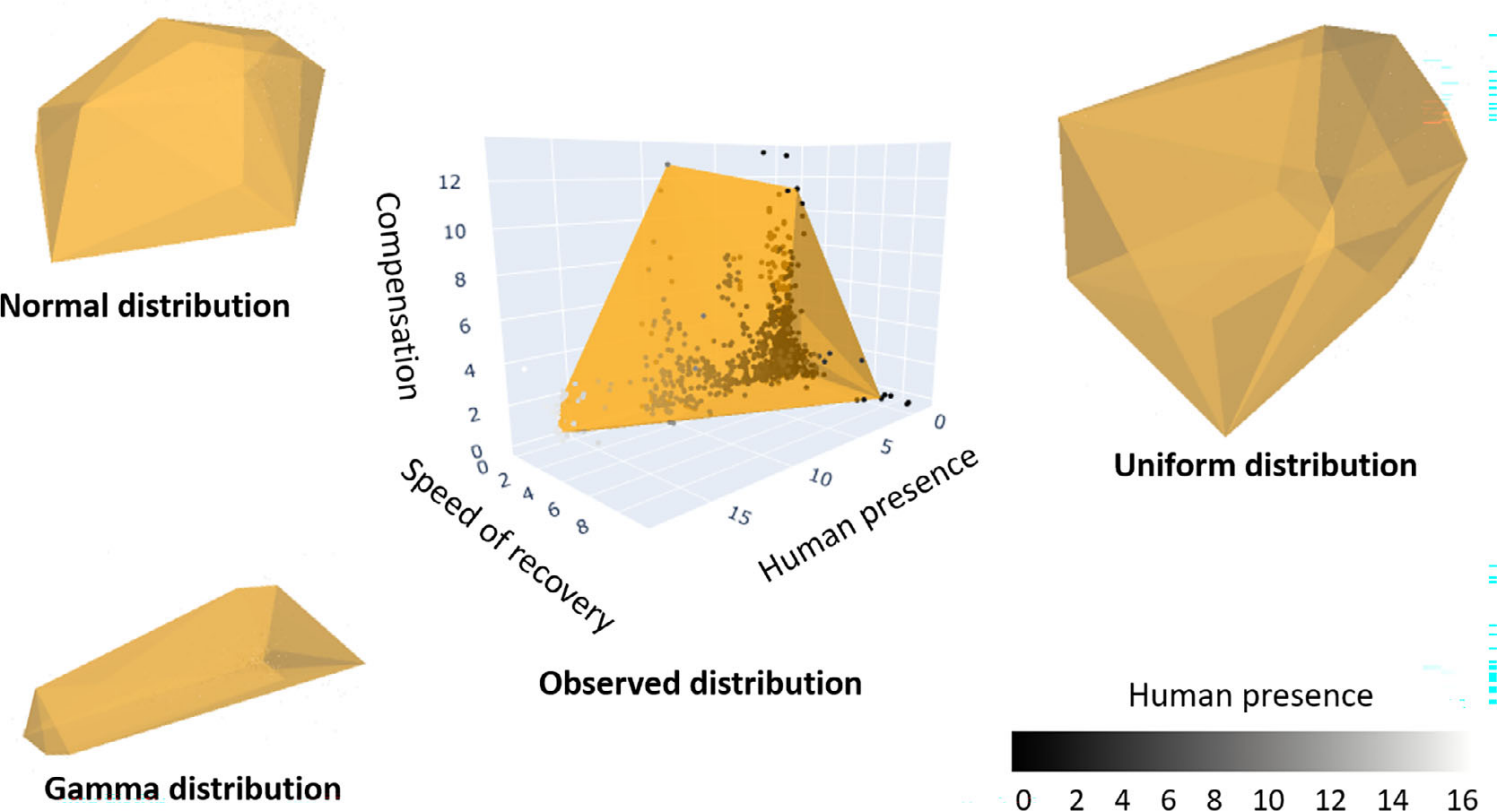
Increases in human presence correlate with lower volume of resilience patterns. Plot of the 3D framework composed of human presence (PC1 in Figure 1), speed of recovery, and compensation, and projection at the same scale of the hypervolume defined by compensation—speed of recovery—human presence under the hypothesis of a uniform, normal, and gamma distribution of the resilience. Due to limitations in graphical representation of a 4D convex hull, here we show the 3D outputs without compensation. An open‐access interactive 3D framework is accessible via https://colab.research.google.com/github/merrienthomas/HFP_appendix/blob/main/display_fig.ipynb and permanently archived in the “display_fig.ipynb” file in Zenodo at https://doi.org/10.5281/zenodo.21397798 (Merrien et al., 2026a).

**TABLE 1:** Simulated data under a uniform, normal, and gamma distribution do not reflect the distribution of the observed data.

Distribution type	Human pressure	Agricultural land use	Overall volume
Uniform			8096.817
Expected volume	1788.540	1060.188	
Observed volume	494.763	311.027	
*p*‐value	1.000	1.000	
% overlap	0.919	0.806	
Normal			3128.046
Expected volume	871.590	515.889	
Observed volume	494.763	311.027	
*p*‐value	1.000	1.000	
% overlap	0.611	0.701	
Gamma			4556.569
Expected volume	1591.067	937.000	
Observed volume	494.763	311.027	
*p*‐value	1.000	1.000	
% overlap	0.824	0.782	
Observed			1855.050

*Note*: Comparison of the resilience hypervolumes of simulated data with the observed data when taking the 4D hypervolumes of the three resilience components (speed of recovery, resistance, and compensation) and human presence or agricultural land use. The last column shows the overall volume of the 5D hypervolume of the three resilience components, human presence, and agricultural land use for simulated data and observed data.

To further explore our finding that the volume of demographic resilience patterns decreases with increasing human activities, we quantified the functional diversity of resilience, measured as resistance, speed of recovery, and compensation. Along the human presence axis, the kernels of the five different levels of human presence exhibit the highest richness at intermediate human presence but a marked contraction in both richness and dispersion at the highest levels of human presence (Figure 4A–C; Appendix S1: Figure S1; Table 2). Although richness peaks at moderate levels of human presence, the most densely populated environments consistently show the lowest richness and dispersion values, supporting the interpretation of resilience thinning at extreme human pressures. The opposite trend emerges for the agricultural land use axis, with increases in the richness and dispersion of resilience patterns in habitats characterized by intensive agriculture and lower building density (Figure 4D–F; Appendix S1: Figure S2). Kruskal–Wallis tests based on the rarefied kernels show that richness (KW‐χ^2^_df=4_ = 36.152, *p* < 0.001; Figure 4A), dispersion (KW‐χ^2^_df=4_ = 32.772, *p* < 0.001; Figure 4B), and evenness (KW‐χ^2^_df=4_ = 20.879, *p* < 0.001; Figure 4C) differ along human activities sections for the different levels of interaction. Similarly, for the agricultural land use axis. For agricultural land use, Kruskal–Wallis tests also show that richness (KW‐χ^2^_df=2_ = 21.677, *p* < 0.001; Figure 4D), dispersion (KW‐χ^2^_df=2_ = 19.726, *p* < 0.001; Figure 4E), and evenness (KW‐χ^2^_df=2_ = 9.951, *p* = 0.007; Figure 4F) differ along this axis. Nonparametric Mann–Whitney *U* tests also show that richness and dispersion of the kernels are lower in habitats with high human presence (scores on human presence axis: 14.5 to >18 in Figure 1) than in habitats with less human activity (human presence axis: <−2 to 2.5) (*p* < 0.001 in both cases). Similarly, in habitats characterized by a high intensity of agricultural land use, nonparametric Mann–Whitney *U* tests show that richness and dispersion of the kernels are higher than in habitats with less agriculture.

**FIGURE 4:**
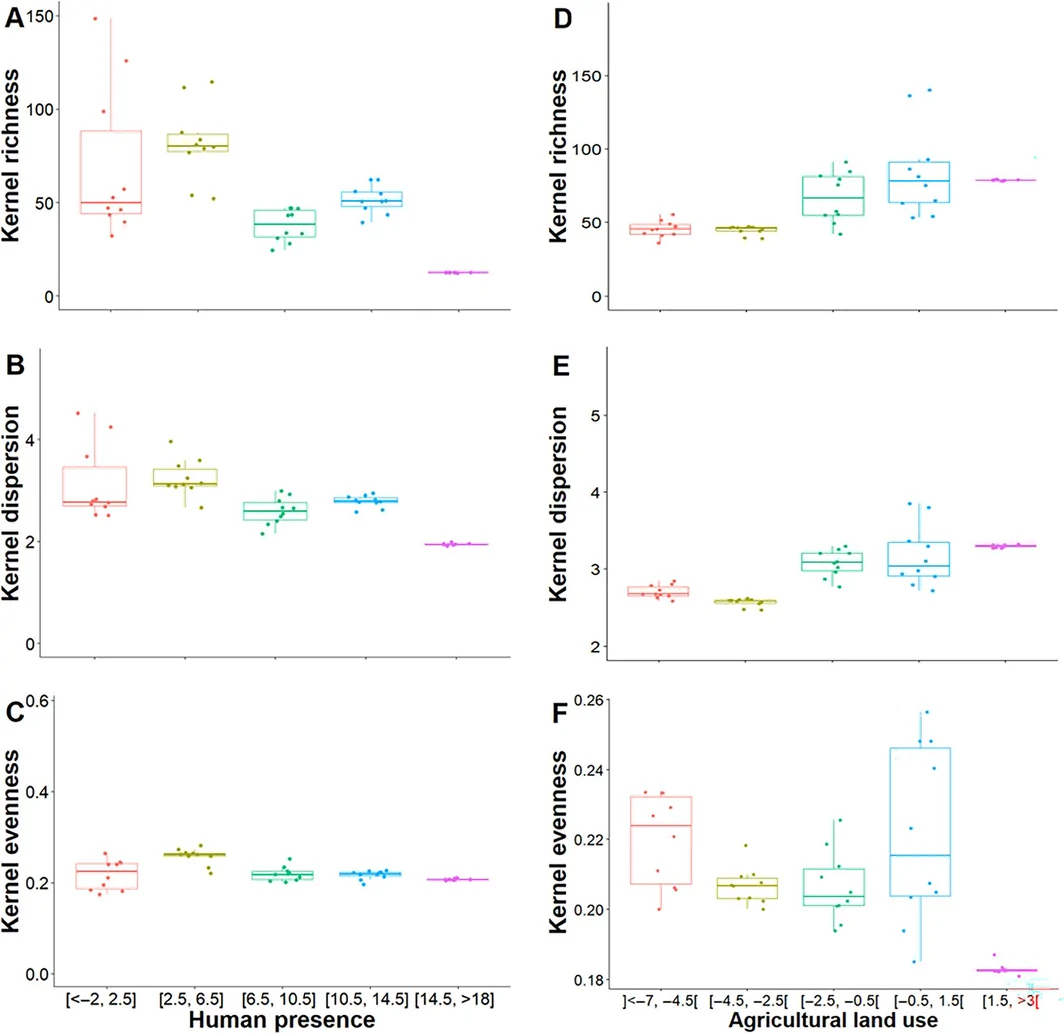
The functional diversity of the three components of demographic resilience is different in areas with high human presence compared to areas with a lower human presence, as well as in areas with a high density of built compared to areas with intensive agricultural land use. The (A) richness and (B) dispersion of the resilience functional diversity, but not its (C) evenness, are lower in areas with high human presence. The (D) richness and (E) dispersion of the resilience functional diversity, but not its (F) evenness, are higher in areas with agriculture compared to densely built areas. Functional diversity plots of the kernels for the different human presence and agricultural land‐use levels after rarefaction analyses. Boxplots represent distributions of kernel richness, dispersion, and evenness across 10 rarefied subsamples per bin.

**TABLE 2:** Richness and dispersion of the resilience kernel decrease when human presence intensifies.

Property	[<−2, 2.5[	[2.5, 6.5[	[6.5, 10.5[	[10.5, 14.5[	[14.5, >18]
Richness	47.542	69.066	34.800	42.533	6.536
Dispersion	3.212	3.332	2.696	2.631	1.521
Evenness	0.003	0.036	0.065	0.129	0.132

*Note*: Richness, dispersion, and evenness values of the kernels for different levels of human presence ([<−2, 2.5[on the PC1 axis of Figure 1 corresponds to low human presence, while [14.5, >18] corresponds to high human presence) without rarefaction analyses.

### Mobility as the fourth dimension of resilience to human disturbances

Using phylogenetically and spatially corrected GLMs to test the effect of mobility on resilience, we find that mobility (as a class variable) does not influence the relationship between either the human presence axis or the agricultural land use axis and any of the three components of demographic resilience (Appendix S1: Table S4). However, an ANCOVA without phylogenetic or spatial corrections reports mobility as correlated with all three components of demographic resilience, whereby animal species with a short mobility have, on average, lower resistance and speed of recovery and higher compensation, compared to animal species with a high mobility (Appendix S1: Table S5). Furthermore, phylogenetically and spatially corrected GLMs of resilience metrics against the two axes of human activities carried out separately for the low‐mobility and high‐mobility species show important correlations in resilience traits (Appendix S1: Table S6). Hence, the damping ratio that is used as a proxy for speed of recovery is significantly lower in habitats with a high human pressure for animal species with a long migration potential, which means that they are faster to recover after a disturbance compared to populations in habitats with less human presence.

### Impacts of human activities on life history traits

Phylogenetically and spatially corrected analyses using collapsed matrices (i.e., all MPMs have 3 × 3 dimensions) report correlations between human activities and key life history traits and their vital rates (Appendix S1: Table S2) that are known to enhance resilience (Capdevila et al., 2020, 2022). The age at first reproduction for plants increases in areas with a higher human presence compared to less densely populated areas (*Z*‐score = 2.130, *p* = 0.033, Appendix S1: Table S2), while sexual reproductive rates decrease in more densely populated areas (*Z*‐score = −2.303, *p* = 0.021). Restricting analyses to studies conducted in 1993 or 2009 does not qualitatively alter the main conclusions. While some plant traits (e.g., age at maturity, recovery, resistance) lost statistical significance along the human presence axis under the reduced sample size (*n* = 291 studies overlapping the 1993–2009 HFP database window), and for animals, net reproductive rate became positively correlated with the said axis, the overall pattern of constrained resilience space near human presence remained unchanged (Appendix S1: Table S7).

## DISCUSSION

In an era characterized by global changes and changes in disturbance regimes (Newman, 2019), examining which, where, and how species are affected by said disturbances is paramount to effective conservation biology (Hobbs & Huenneke, 1992). As human activities are an important cause of disturbance regimes (Battisti et al., 2016), here we examine how different dimensions of human activities correlate with the demographic resilience of hundreds of plant and animal species. By applying phylogenetically and spatially explicit regressions to 956 wild populations worldwide, we find that species inhabiting areas with higher human activities are not more resilient than those in more pristine habitats, contrary to our initial expectations. However, we find that the spectrum of resilience patterns these species display gets thinner towards areas with high human activity compared to areas with fewer anthropic activities. As such, we show a decay in the “demographic resilience portfolio” in areas characterized by high human activity. Furthermore, we detect no link between the ability of species to move long distances (thus potentially avoiding local/regional disturbances) and resilience in the 47 animal species of our dataset.

Our key finding, that natural populations closer to human settlements display a more constrained repertoire of demographic resilience strategies, can be interpreted in two non‐mutually exclusive ways. First, species with very high or very low resistance, compensation, or recovery speed may have been selectively filtered out of highly disturbed habitats via local extinction, reflecting a failure to cope with anthropogenic pressures (e.g., sensitive mammal taxa show reduced richness with increasing human density, consistent with extinction filtering [Greco et al., 2025]). Second, the observed pattern may arise from species sorting, whereby only a subset of species with particular demographic profiles occurs in human‐dominated landscapes (e.g., disturbance‐tolerant bird species dominating urban and rural sites relative to more natural habitats [Villegas Vallejos et al., 2016]). Importantly, an alternative and complementary explanation is that species exhibiting extreme resilience strategies may be associated with environmentally harsh, climatically variable, or low‐productivity systems that humans have historically avoided for settlements. Humans have disproportionately settled in areas of relatively high primary productivity, moderate climates, and environmental stability (e.g., Ellis et al., 2010; Foley, 2005), which may not overlap with the habitats where highly disturbance‐adapted life histories evolved. Under this scenario, the reduced representation of extreme resilience strategies near human settlements would not necessarily reflect an inability to cope with anthropogenic disturbance, but rather a spatial mismatch between human land use and the environmental contexts that select for such demographic strategies. Because most species in our dataset are represented by a single population, even though our analyses incorporate spatial and phylogenetic corrections, we are cautious about attributing these patterns to contemporary local adaptation or evolutionary processes. Rather, our results are consistent with macroecological filtering or sorting processes operating across human‐modified gradients.

The examined volume of demographic resilience suggests that species with the most extreme values of demographic resilience in our study (e.g., very high or very low values of compensation) do, in fact, not thrive in urban environments. This is an interesting result, as urban habitats are typically characterized by frequent disturbances, though of relatively low intensity (Blair, 1996; Rebele, 1994). A plausible explanation for our finding is therefore that highly resilient species might not have been present in areas where humans settled hundreds/thousands of years ago. Indeed, humans have historically tended to settle around high primary productivity and relatively stable climate areas (Durand‐Dastes, 1986). By offering stable environmental conditions, these locations might not have favored adaptations to disturbances (Cant et al., 2022; Holling, 1973). Another explanation would be that species with extreme values of resilience (very high or very low) have been excluded from these locations. Because resilience traits can be costly to maintain when disturbances are infrequent or mild (Osier & Lindroth, 2006), urban habitats characterized by relatively low‐intensity disturbances may not favor their persistence (Blair, 1996; Rebele, 1994). Indeed, maintaining some degree of resilience often comes at the expense of other vital functions and life history traits, and can, for instance, lead to a reduced competitive ability in stable environments (Melnyk et al., 2015). Overall, the thinning of the spectrum of resilience patterns along the axes of human activities adds support to evidence for the human‐induced biotic homogenization of natural systems (McKinney & Lockwood, 1999), whereby the diversity of resilience patterns is expected to decrease in areas modified by human activities, such as cities and intensively farmed lands.

Our work suggests a potential effect of human activities on the distribution of resilience across the globe. However, our method cannot demonstrate whether or not evolutionary processes leading to a global homogenization of resilience are at stake. Recent work from Cant et al. (2023) showed that exposure to recent environmental stochasticity—a form of disturbance regime—does not necessarily result in an increase in the intrinsic resilience of wildlife populations. Their results highlight the difference in timescale between adapting to and experiencing disturbances. Going forward, it would be of high interest to investigate the potential evolutionary pressures of disturbance regimes on natural populations through common garden experiments (Berend et al., 2019; de Villemereuil et al., 2016; Schwinning et al., 2022). On a broader scale, in a world where environmental stochasticity has been increasing in the last century (Lenton et al., 2017), understanding the evolutionary dynamics of demographic resilience in populations will likely support more cost‐effective conservation plans (Capdevila et al., 2020).

Although our data do not provide evidence for a link between mobility, human activities, and resilience, we argue that this hypothesis should not be neglected. Of relevance in this context is the finding by Tucker et al. (2018), who found that mammals in areas of high human activities have a much more constrained movement range than those in areas characterized by lower human activities. Hence, even though here we report an inconclusive trend between mobility and demographic resilience for 47 animal species, the correlation between human presence and some aspects of resilience when separating species with long‐distance and short‐distance mobility points toward the need to better understand whether mobility could indeed be the fourth dimension of demographic resilience, together with resistance, speed of recovery, and compensation. Indeed, both climate change and human activities can lead to changes in species' ranges and extinction risks (di Marco et al., 2019; Pacifici et al., 2020; Rubenstein et al., 2023; Zhang et al., 2020). Hence, it is of high interest to understand how mobility, migration distance, and resilience correlate and interact to produce more comprehensive conservation policies.

Because generation time is linked to resistance and speed of recovery in both plants and animals (Capdevila et al., 2022), we decided to investigate the correlation between life history traits, other demographic traits, and human activities. We found that plant species in densely human‐populated areas achieve sexual maturity later than plant populations in more pristine areas. All else being equal, reaching maturity at a younger age allows populations to better deal with habitat disturbances by producing offspring earlier and hence having more generations per unit of time (Traill et al., 2014). Densely human‐populated areas would then offer more stable conditions, as humans tend to settle in more stable areas (Durand‐Dastes, 1986), reducing the pressure on reproductive traits. These key findings provide support for our expectation that species' life history traits and their underlying vital rates modulate the relationships between human activities and their demographic resilience. Moreover, even though we found no clear evidence that human activities impact reproductive rates of animals, such effects have been reported in other studies where human activities led to higher breeding densities in birds (Brown & Graham, 2015; Chamberlain et al., 2009), mammals (French et al., 2011; Lewis et al., 2021), and plants (Zhao et al., 2023). However, we note that for the 300 plant species examined here, our results show a decrease in sexual reproduction in more densely populated areas, showing a contrast between the overall reproduction and the sexual reproduction.

Our macroecological approach, based on structured population data of an unprecedented number of wildlife populations, highlights the promise that metrics of transient performance of ecological systems hold for our predictive ability of demographic resilience. Our resilience framework, founded on transient demographic metrics (Capdevila et al., 2020; Neubert & Caswell, 1997), allows for the exploration of the range of potential immediate responses of species populations to disturbances. Surprisingly, these metrics are not yet commonly used in population ecology (Cant et al., 2022; Capdevila et al., 2020; McDonald et al., 2016). Despite this gap in knowledge, these metrics remain a powerful instrument to inform conservation programs and species risk assessments (Iles et al., 2016; Jelbert et al., 2019).

Here, we demonstrate that the resilience patterns of 956 wildlife populations become less diverse as human activities increase. This loss of variation in resilience patterns, we argue, is an overlooked form of biodiversity loss, as it likely translates into the loss of “adaptive potential” (Nielsen et al., 2023). This loss presents a tangible risk to the viability of species less able to cope with the diversity of disturbances that characterize the Anthropocene (Pörtner & Roberts, 2022). Altogether, our findings point out that land use and human activities are not only a strong predictor of species' evolution (Baltazar‐Soares et al., 2021), geographic range (di Marco & Santini, 2015), and extinction rates in mammals (Andermann et al., 2020) and plants (Darrah et al., 2017) but also of the diversity of resilience patterns of species at global scales. These proxies of demographic resilience, solidly based on transient metrics, quantify the “transient envelope” of *potential* responses of natural populations to multiple kinds of disturbances (Capdevila et al., 2020; Stott et al., 2011) that shape the structure of a population, commensurate with the a/biotic conditions that have shaped the values of the demographic data collected from the field populations. Future work must examine how these metrics operate under the realistic constraints of density dependence (Hernandez et al., 2025), and the region‐specific disturbances that each population is most likely affected by (Carnicer et al., 2021), as well as the links to the community context of each population, a glaring gap in knowledge in the field (Sutherland et al., 2013). Another promising avenue of research is the examination of intraspecific variation in demographic resilience, for which the employed databases do not yet have sufficient spatial replication (Römer et al., 2021), and for which work with other species is currently underway (Smith et al., 2020). Species such as *Plantago lanceolata* (Smith et al., 2020) or *Bombina variegata* (Cayuela et al., 2022) that offer in other databases a high number of spatial (>60 populations ea.) and time replicates of demographic data would be of great help to better understand the mechanisms that lead to variable patterns of resilience.

Together, our findings emphasize the need for an integrative approach to unify macroecological approaches with long‐term monitoring and experimental approaches to understand and predict the resilience of species across the Tree of Life in the Anthropocene. We hope that these key findings will be integrated into forecasts of wildlife populations and the assessment of species in the IUCN Red List (IUCN, 2023).

## AUTHOR CONTRIBUTIONS

Roberto Salguero‐Gómez developed the concept, led efforts to archive the demographic data and phylogenies, and guided every component of the manuscript. Roberto Salguero‐Gómez developed the hypotheses with input from Thomas Merrien and Katrina J. Davis. Moreno Di Marco provided the human activity data. Thomas Merrien and Roberto Salguero‐Gómez carried out the analyses with input from Katrina J. Davis and Pol Capdevila. Roberto Salguero‐Gómez and Thomas Merrien wrote the article, with critical input from all authors. All authors discussed the results and implications and commented on the manuscript at all stages.

## CONFLICT OF INTEREST STATEMENT

The authors declare no conflicts of interest.

## Supporting information


Appendix S1.

## Data Availability

Data (Merrien et al., 2026b) are available in Zenodo at https://doi.org/10.5281/zenodo.22649459. Code (Merrien et al., 2026a) is available in Zenodo at https://doi.org/10.5281/zenodo.21397798.
