# Supplementary material for: The diversity of resilience patterns of natural populations decreases closer to human activities
Source: Ecology. 2026 Nov 5;107(11):e70557. doi: 10.1002/ecy.70557 (PMC13740652; doi:10.1002/ecy.70557)
Supplement: Supplementary file 1 [file ECY-107-e70557-s001.pdf]

Appendix S1

The diversity of resilience patterns of natural populations decreases closer to human activities

Thomas Merrien, Katrina J Davis, Moreno Di Marco, Pol Capdevila, Roberto Salguero-Gómez

Ecology

This file includes:

Supporting text
Figures S1 to S6
Tables S1 to S12
SI References

Supporting information text

Extended methods

Calculation of demographic traits. From the selected 956 MPMs, we extracted vital rates, life history traits, and transient dynamics metrics to study how they correlate with different levels of human activities. In COMPADRE and COMADRE, the majority of MPMs ***A*** have been divided into 3 submatrices ***U***, ***F*** and ***C***, such that ***A*** = ***U*** + ***F*** + ***C***. The submatrix ***U*** contains survival-dependent demographic processes, such as progression and retrogression from one stage to another; the submatrix ***F*** contains sexual per-capita contributions; the submatrix ***C*** contains clonal per-capita contributions.

Among the key traits to understand species' demographic strategies, vital rates allow us to describe precisely how species allocate resources across the different life stages. Vital rates have the advantage of being easily extracted from the MPMs. To extract the vital rates of progression (moving onto larger, more developed, and/or older stages in the life cycle of the species), shrinkage (moving backwards), per-capita sexual reproduction, and per-capita clonal reproduction of stage *j*, we summed the elements of the jth column corresponding to posterior stages of the matrix ***U***, the elements of the jth column corresponding to anterior stages of the matrix ***U***, all the elements of the jth column of the matrix ***F***, and all the elements of the jth column of the matrix ***C***, respectively. We then averaged the vital rates from each stage weighted by their contribution at the demographic equilibrium to obtain a population-level vital rate estimate. This contribution of each stage at the demographic equilibrium (*i.e.*, the distribution of the different stages at stationary equilibrium), is summarised by the stable stage distribution vector ***w***, the right eigenvector of ***A*** (Caswell 2000). This vector ***w*** represents the proportion of individuals in each stage when the population is at the stationary equilibrium. The vital rate of survival of stage *j* is obtained by averaging the sum of all the columns of the matrix ***U*** weighted by their contribution as per ***w*** above. As an illustration, to calculate the vital rate of sexual reproduction and progression the

formulae are as following: $\sum_j \; (\sum_i \; f_{ij})w_j$, and $\sum_j \; (\sum_{i>j} \; u_{ij})w_j$, respectively, with f_{ij} being the element corresponding to the ith *row* of ***F*** in column j, and u_{ij} being the element of ***U*** in column j, row i.

Data global representation. Plotting the different localities of the 956 studied populations showcases their quasi-global distribution, though not exempt of important biogeographic biases (Fig. S1). The vast majority of the demographic data archived in COMPADRE and COMADRE come from peer-reviewed publications following the dynamics of wildlife populations around the world. However, most of the studies present in these open-access resources are located in North America and Europe. Asia, Oceania, and South America are currently poorly covered(Amano *et al.* 2016).

Phylogenetic information. Phylogenetic relatedness can generate biases in comparative analyses (Felsenstein 1985; Harvey & Pagel 1991). Moreover, in our dataset, several species contain more than one population [Table S1; "list_plant_species.pdf" in https://doi.org/10.5281/zenodo.22649459 (merrienthomas 2026)], requiring that our analyses include a hierarchical treatment of the data. Therefore, we collected phylogenetic information at the species level to implement a series of phylogenetically corrected analyses. The phylogenetic tree of animals was obtained via TimeTree (Kumar *et al.* 2017) and the one for plant was generated using V.phylomaker (Jin & Qian 2019). For the animal phylogeny, three species were missing using TimeTree, so they were included by examining in the literature and identifying the distances that separate them from their closest relatives in our tree. To do so, we used the Open Tree of Life (OpenTreeofLife *et al.* 2019; OpenTreeOfLife *et al.* 2019).

Demographic trait regression. To validate the outputs of our “phyr-pglm” and “MCMC-pglm” models, we ran a set of additional models. As working on residuals can be controversial because one might neglect important pieces of information

(Chen *et al.* 2018; Freckleton 2002) —namely that model estimates based on residuals can provide biased estimates and this approach also increases the significance of variables that are more sensitive to this method—we ran models with the same original data but without using residuals. We included size as an explanatory factor when it was relevant (Fisher's exact test) and ran a Markov Chain Monte Carlo generalized linear mixed model (MCMCglmm) to account for evolutionary history and a Generalized Additive Models (gam) model with longitude and latitude to account for spatial distribution.

Extended results

Linear model in the resilience framework. Plant populations inhabiting places with a high human presence are on average more resistant that populations in areas with less human presence. Table S5 shows the results of the linear model correlating the different components of resilience and human activities characterized by PC1 and PC2. The table shows that, for plants, resistance is significantly higher for populations closer to human presence. For animal populations, resistance significantly decreases with agricultural land use, meaning that areas with intensive agriculture on average host less resistant animal species.

Phylogenetically corrected regressions in the resilience framework. We used phylogenetically and spatially corrected models to test the correlation between the different components of demographic resilience and axes of human activities (Table S6). We first ran gam models accounting for spatial heterogeneity in our dataset and used the residuals of the gam in phylogenetically corrected glmms using the *phyr* R package (Li *et al.* 2020) as described in the methods section.
These results show how habitats characterised by high human activities, despite not hosting the most resilient species, host species with on average more resistant, faster recovery, but lower propensity to compensate, and therefore overall higher resilience than less human-disturbed habitats.

Further demographic traits analyses. We ran phylogenetically and spatially corrected analyses for the sub-selection of animal species to study the different mobility level (*i.e.* high *vs.* low mobility), as outlined in H3 in the main manuscript. Comparing species with high mobility *vs.* low mobility, human activities were found to affect them differently. For instance, populations with a low mobility are more resistant in dense agricultural areas compared to their counterparts located in pristine habitats. This trend is, however, not observed in populations with a high mobility.

Populations with a low mobility have a lower age at first reproduction in disturbed habitats, while species with a high mobility have a shorter generation time in habitats with high human activities. These results emphasize the importance of mobility to understand the responses of animal populations to human disturbances.

Figures

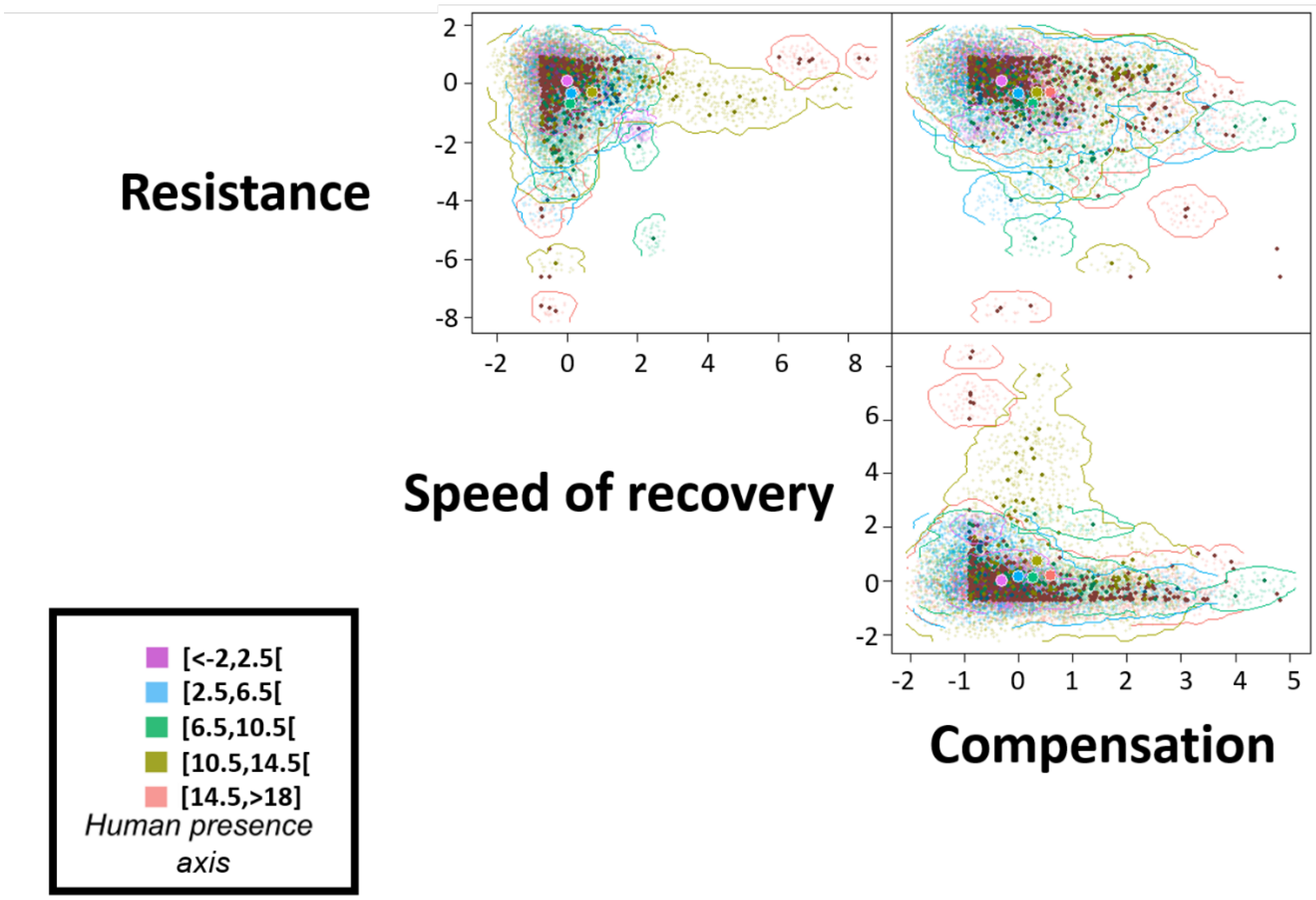

Fig. S1. The kernels of the three resilience components shrink before increasing levels of human activity. Pair-wise kernel representation of the three components of demographic resilience as a function of five pre-set boundaries based on the scores of the human presence axis (PC1 in Fig. 1). The coloured dots identify the centroid of the associated kernel.

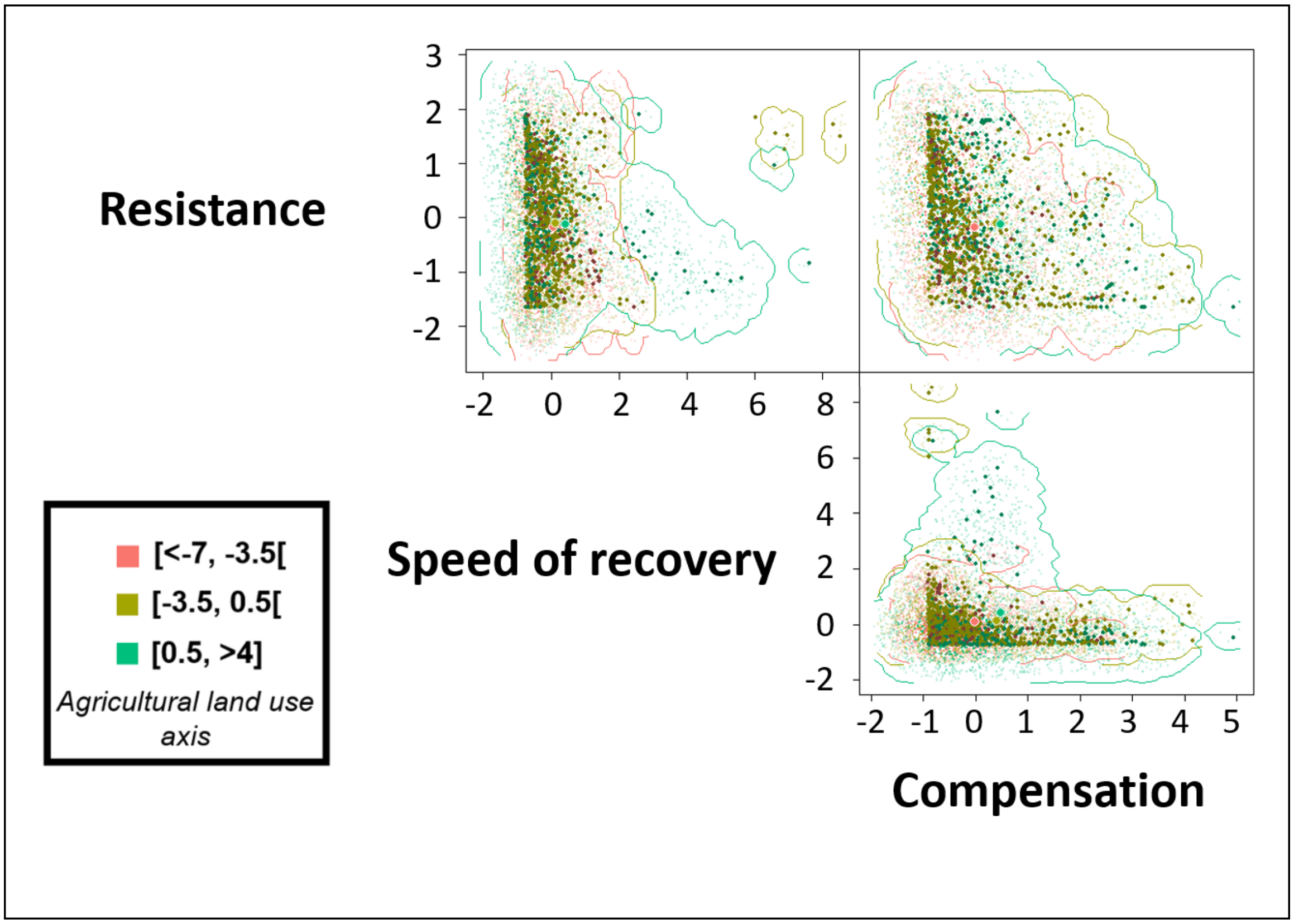

Fig. S2. The kernel of the resilience components in areas with more intensive agricultural land use is wider than in areas with less intensive agricultural land use. Pair-wise kernel representation of the three components of demographic resilience as a function of five pre-set boundaries based on the scores of the agricultural land use axis (Fig. 1). Red colour corresponds to areas with less intensive agricultural land use, green colour to intermediate areas and blue colour to most intensive agricultural land use. The coloured dots identify the centroid of the associated kernel.

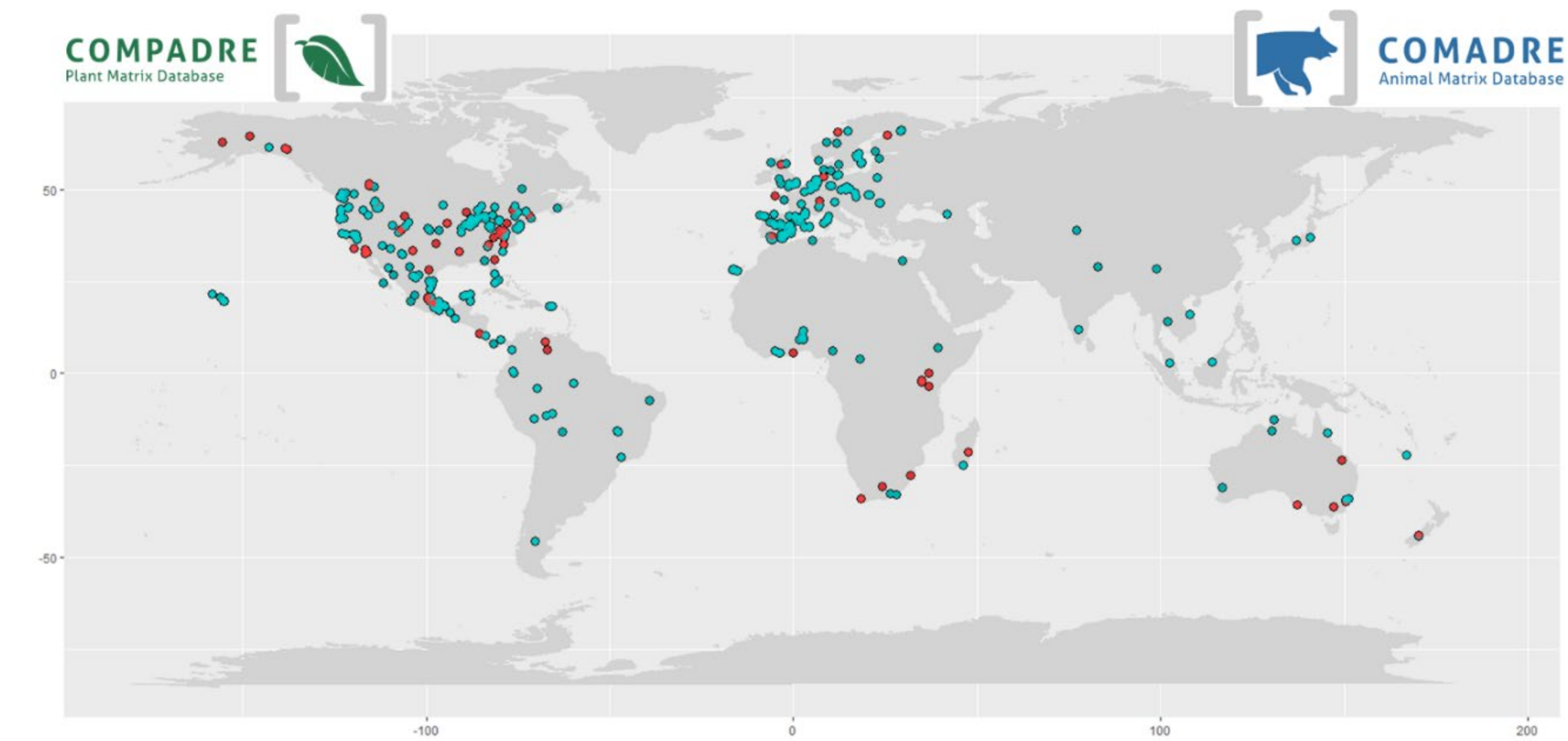

Fig. S3. Global distribution of the 956 wild populations used in this study. Light blue points represent studied plant populations, while light red ones represent animal populations. Some geographic areas are not well represented, such as Asia, Africa, or South America.

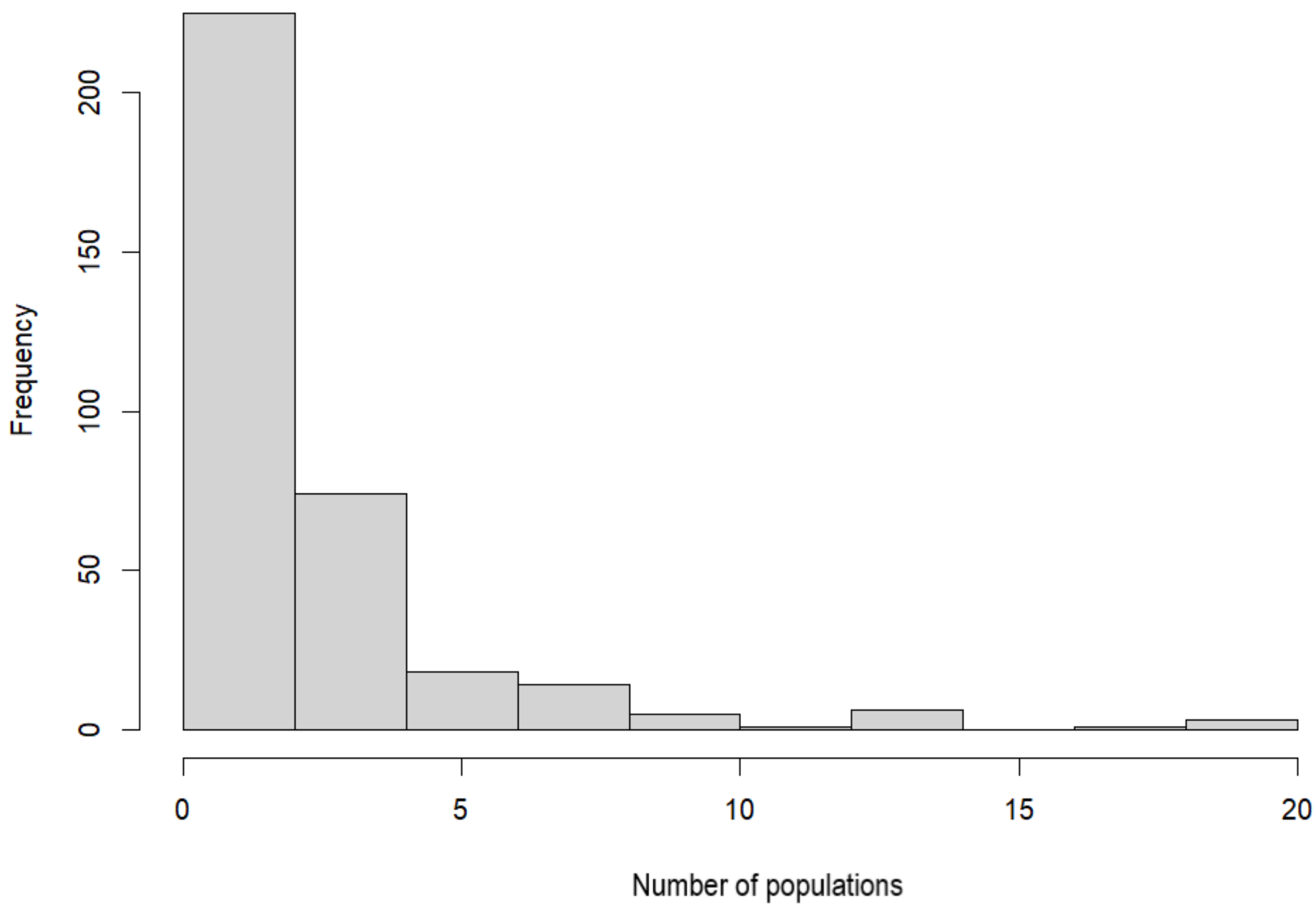

Fig. S4. Histogram of the distribution of the number of populations per species in our dataset.

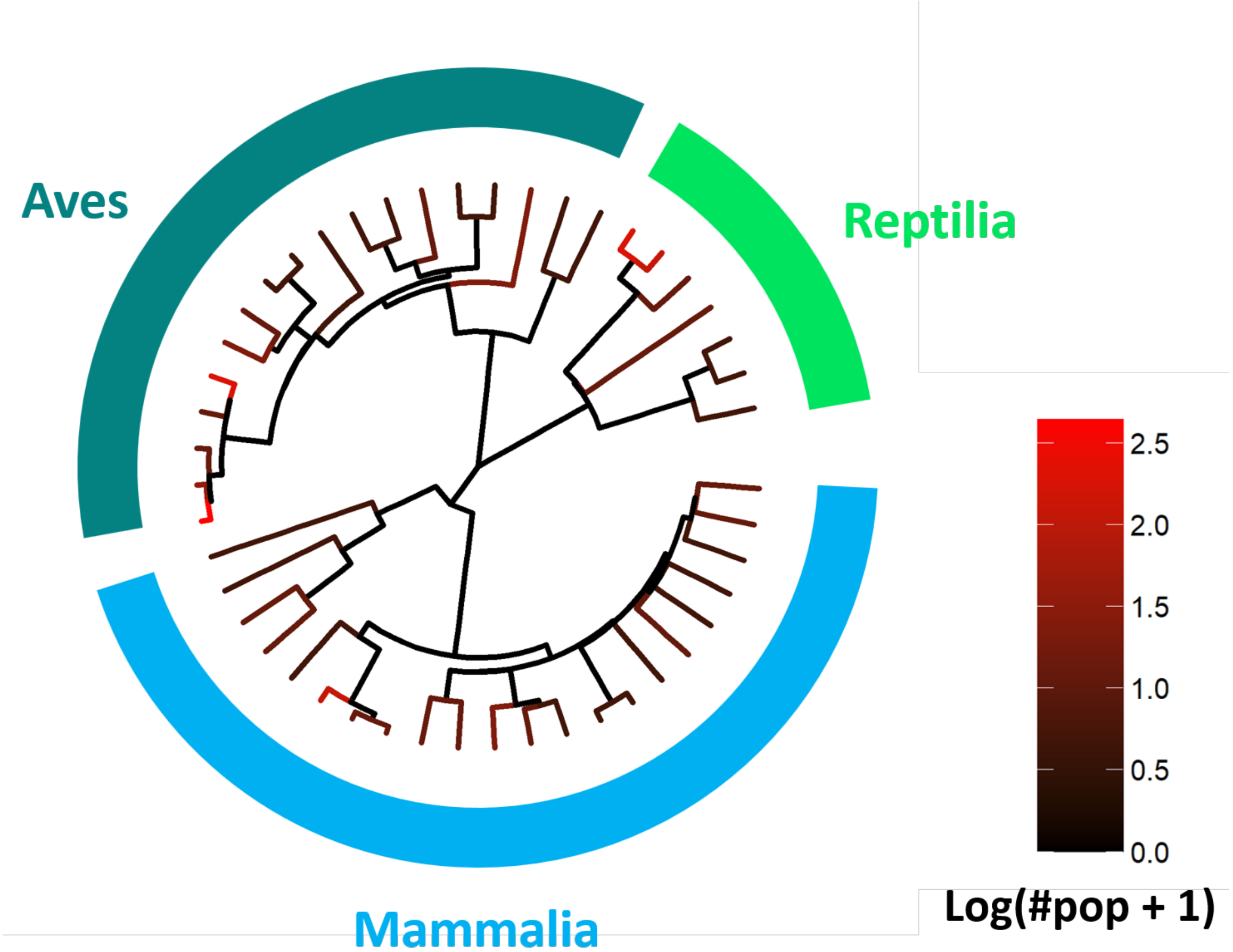

Fig. S5. Resolved species-level phylogenetic tree of the 112 populations within 47 animal species used in our analyses. The phylogeny was obtained using Timetree (Kumar *et al.* 2017) and modified to color the species tips according to the number of populations per species (in log scale) (Table S1). The legend shows the color gradient corresponding to the number of populations per species (in log scale).

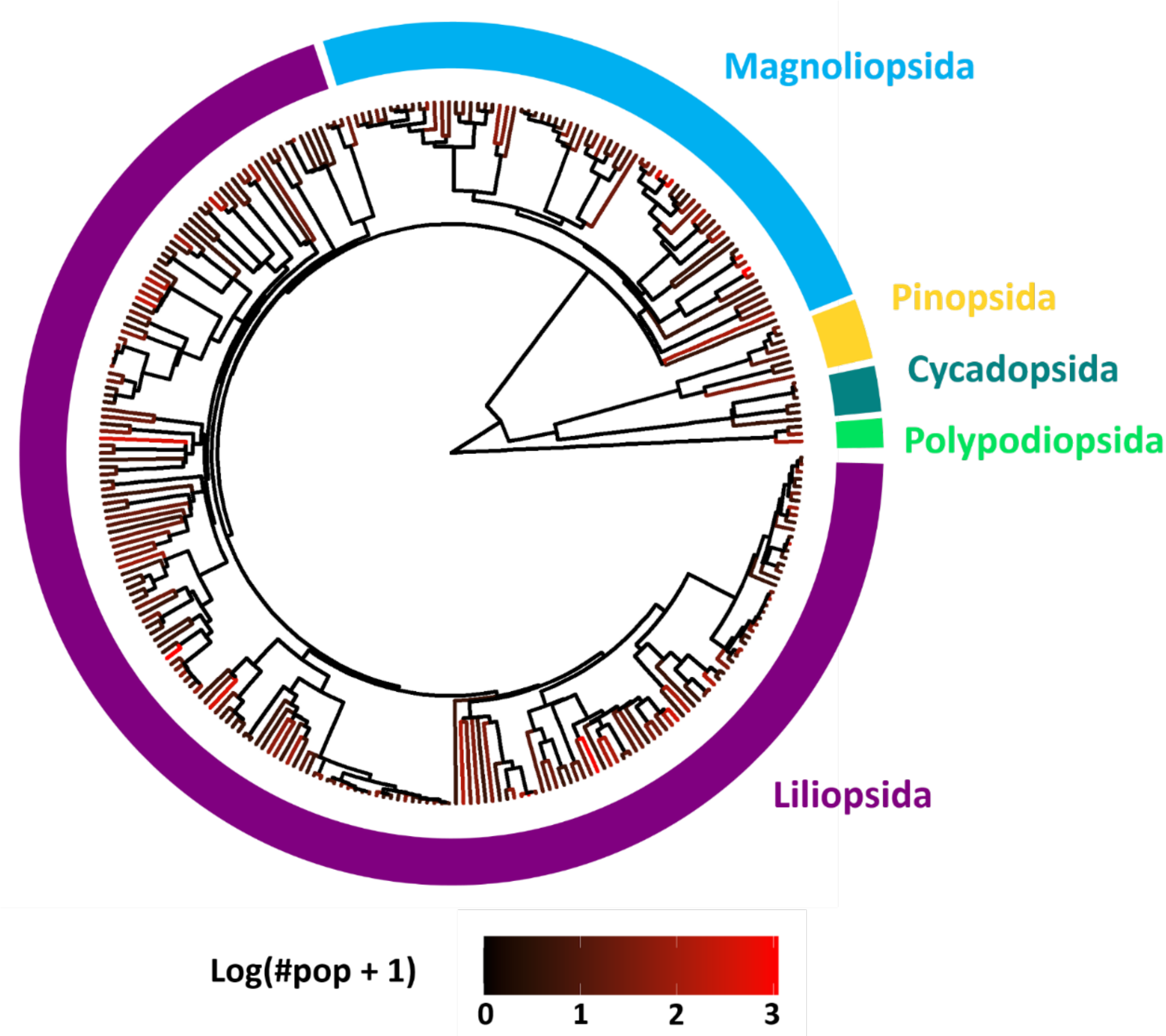

Fig. S6. Resolved species-level phylogenetic tree of the 844 populations within 300 plant species used in our analyses. The phylogeny was obtained using V.Phylomaker (Jin & Qian 2019) and modified to color the tip edges according to the number of different populations per species (in log scale) ["list_plant_species.pdf" in https://doi.org/10.5281/zenodo.22649459, (merrienthomas 2026)]. The taxonomic class to which the populations belong is shown around the tree. The legend shows the color gradient corresponding to the number of populations per species (in log scale).

Tables

Table S1. Three components of the principal component analysis (PCA) of the Human Footprint Database (HFP (Venter *et al.* 2016)), were statistically significant. Here, we retain the first two due to their ease for biological interpretation (Fig. 1). Summary of the PCA with the eight layers of Human Footprint from Venter and collaborators (Venter *et al.* 2016). The eight layers of the Human Footprint are: human population density, roads, navigable waterways, railways, light pollution, built environment, extensive pastures, and intense croplands (Fig. 1). The table shows the eigenvalue associated with each PC, as well as the percentage of variance explained.

Principal component axis	Eigenvalue	Percentage of variance explained
PC 1	2.869	35.862
PC 2	1.330	16.626
PC 3	0.981	13.723
PC 4	0.963	12.038
PC 5	0.681	8.516
PC 6	0.562	7.029
PC 7	0.417	5.213
PC 8	0.079	0.992

Table S2. Summary of the results of the trait analysis for plants (light blue) and animals (light red) using the spatially and phylogenetically corrected model with corrections for matrix dimension. The human presence axis and agricultural land use axis refer to the two main dimensions of human activities from the Human Footprint database, as detailed in Figure 1. Results are provided according to the following format: for each axis of the human pressure, the estimates of the effect size, the standard error, the Z-score and the associated p-value bolded when below 0.05. Note that none of the examined animal populations include shrinkage or clonal reproduction (thus "-").

"pglmm" method for phylogenetically and spatially corrected model for plants and animals with collapsed matrices								
	**Human presence**				**Agricultural land use**			
Trait	*Estimate*	*S.E.*	*Z-score*	*P*	*Estimate*	*S.E.*	*Z-score*	*P*
**Vital rates**								
Survival	0.008	0.006	1.469	0.142	0.010	0.009	1.090	0.276
	0.015	0.008	1.816	0.069	0.022	0.016	1.414	0.157
Growth	-0.010	0.006	-1.672	0.094	-0.011	0.009	-1.219	0.223
	0.013	0.008	1.623	0.105	0.025	0.015	1.691	0.091
Shrinkage	-0.002	0.002	-0.896	0.370	-0.001	0.004	-0.291	0.771
	-	-	-	-	-	-	-	-
Sexual reproduction	-0.131	0.057	-2.303	**0.021**	-0.168	0.090	-1.862	0.063
	-0.016	0.043	-0.371	0.711	-0.028	0.083	-0.336	0.737
Clonal reproduction	0.002	0.001	1.762	0.078	0.001	0.002	0.722	0.470
	-	-	-	-	-	-	-	-
**Life history traits**								
Net reproductive rate (R_0)	-0.043	0.067	-0.646	0.518	-0.107	0.106	-1.005	0.315
	0.094	0.061	1.527	0.127	0.154	0.119	1.295	0.195
Degree of iteroparity (S)	0.012	0.020	0.594	0.553	-0.004	0.034	-0.104	0.917
	0.002	0.022	0.083	0.934	0.020	0.043	0.470	0.638
Generation time (T)	0.025	0.030	0.834	0.404	0.074	0.050	1.486	0.137
	0.006	0.025	0.250	0.803	0.015	0.048	0.315	0.753
Age at maturity (L_α)	0.025	0.010	2.445	**0.014**	0.024	0.016	1.505	0.132
	-0.017	0.019	-0.897	0.370	-0.021	0.037	-0.573	0.567
Rate of actuarial senescence (H)	0.006	0.007	0.856	0.392	0.003	0.012	0.240	0.810
	-0.013	0.007	-1.721	0.085	-0.021	0.014	-1.470	0.141

“pglmm” method for phylogenetically and spatially corrected model for plants and animals with collapsed matrices								
	**Human presence**				**Agricultural land use**			
Trait	*Estimate*	*S.E.*	*Z-score*	*P*	*Estimate*	*S.E.*	*Z-score*	*P*
**Transient dynamics**								
Damping ratio (ρ)	0.009	0.005	1.945	0.052	0.006	0.008	0.779	0.436
	0.009	0.109	0.080	0.936	-0.242	0.210	-1.151	0.250
Reactivity ($\bar{\rho}_1$)	0.012	0.007	1.844	0.065	0.015	0.011	1.347	0.178
	0.010	0.008	1.197	0.231	0.010	0.016	0.625	0.532
First step attenuation ($\underline{\rho}_1$)	0.011	0.006	2.038	**0.042**	0.011	0.009	1.223	0.221
	0.014	0.012	1.238	0.216	0.031	0.022	1.403	0.160
Maximum amplification ($\bar{\rho}_{max}$)	-0.043	0.025	-1.741	0.082	-0.026	0.039	-0.665	0.506
	0.010	0.008	1.196	0.232	0.010	0.016	0.656	0.512

Table S3. Correlation between transient metrics and both human activities and generation time (T) with a subset of our data. For each transient dynamic metric, we ran two analyses: one with both the two axes of human activities and generation time, and one with only the two axes of human activities as response variables. The human presence axis and agricultural land use axes refer to the two main dimensions of human activities from the Human Footprint database, as detailed in Figure 1 of the manuscript. Results are provided according to the following format: for each axis of the human pressure, the estimates of the effect size, the standard error, the Z-score and the associated p-value in bold when below 0.05.

**Plants**													
		Human presence (PC1)				Agricultural land use (PC2)				Generation time (T)			
		Estimate	S.E.	Z-score	P	Estimate	S.E.	Z-score	P	Estimate	S.E.	Z-score	P
Damping ratio (ρ)	With generation time (T)	0.001	0.014	0.066	0.948	-0.006	0.024	-0.254	0.799	0.000	0.000	-0.220	0.826
	Without T	0.001	0.014	0.072	0.943	-0.006	0.024	-0.251	0.802	NA	NA	NA	NA
Period of oscillation (*Pi*)	With T	0.006	0.022	0.262	0.793	0.047	0.037	1.244	0.213	0.001	0.000	4,704	**<0.001**
	Without T	0.003	0.022	0.119	0.905	0.044	0.038	1.141	0.254	NA	NA	NA	NA
Reactivity ($\underline{\rho}_1$)	With T	-0.066	0.032	-2.071	**0.038**	-0.077	0.051	-1.528	0.127	0.000	0.000	-0.151	0.880
	Without T	-0.066	0.032	-2.073	**0.038**	-0.077	0.051	-1.529	0.126	NA	NA	NA	NA
First step attenuation ($\underline{\rho}_1$)	With T	0.009	0.005	1.880	0.060	0.006	0.008	0.776	0.438	0.000	0.000	-2,633	**0.008**
	Without T	0.010	0.005	1.936	0.053	0.007	0.008	0.818	0.413	NA	NA	NA	NA
Maximum amplification ($\underline{\rho}_{max}$)	With T	-0.057	0.032	-1.799	0.072	-0.063	0.051	-1.249	0.212	0.000	0.000	-0.882	0.378
	Without T	-0.057	0.032	-1.774	0.076	-0.062	0.051	-1.229	0.219	NA	NA	NA	NA

(Table S3 continued)													
**Animals**													
		Human presence (PC1)				Agricultural land use (PC2)				Generation time (T)			
		Estimate	S.E.	Z-score	P	Estimate	S.E.	Z-score	P	Estimate	S.E.	Z-score	P
Damping ratio (ρ)	With T	0.002	0.007	0.255	0.799	0.004	0.013	0.305	0.761	0.000	0.001	0.167	0.868
	Without T	0.002	0.006	0.300	0.764	0.004	0.013	0.338	0.736	NA	NA	NA	NA
Period of oscillation (*Pi*)	With T	-0.025	0.030	-0.820	0.412	-0.024	0.059	-0.407	0.684	0.037	0.009	4.083	**<0.001**
	Without T	0.003	0.033	0.086	0.932	0.035	0.033	0.554	0.580	NA	NA	NA	NA
Reactivity ($\underline{\rho}_1$)	With T	-0.007	0.009	-0.767	0.443	-0.008	0.018	-0.458	0.647	0.003	0.002	1,564	0.118
	Without T	-0.005	0.009	-0.654	0.573	-0.004	0.018	-0.243	0.808	NA	NA	NA	NA
First step attenuation ($\underline{\rho}_1$)	With T	0.011	0.010	1,013	0.311	0.021	0.021	0.974	0.330	-0.002	0.002	-0.631	0.528
	Without T	0.012	0.010	1.228	0.219	0.026	0.019	1.344	0.179	NA	NA	NA	NA
Maximum amplification ($\underline{\rho}_{max}$)	With T	-0.023	0.018	-1.298	0.194	-0.034	0.036	-0.947	0.346	0.002	0.004	0.490	0.624
	Without T	-0.017	0.017	-0.950	0.342	-0.012	34269.000	-0.350	0.727	NA	NA	NA	NA

Table S4. Mobility does not affect the correlation between demographic resilience and human activities when correcting for spatial and phylogenetic autocorrelations. Results of the "phyr-pglm" models of the different components of the resilience framework against the two axis of human activities (PC1 and PC2; see Fig.1) and mobility. Species are classified as high mobility (range > 100 km) *vs*. low mobility (< 100 km). Bolded P-values are statistically significant at P < 0.05.

	Value	Z-score	P-value
Resistance			
PC1	0.006	0.239	0.811
PC2	0.044	0.809	0.419
Mobility (low)	0.432	-0.141	0.888
Speed of recovery			
PC1	0.006	0.243	0.808
PC2	-0.011	-0.216	0.829
Mobility (low)	-0.247	-1.273	0.203
Compensation			
PC1	-0.051	-2.239	**0.025**
PC2	-0.109	-2.153	**0.031**
Mobility (low)	-0.141	-0.400	0.689

Table S5. Mobility modulates the correlation between demographic resilience and human activities when spatial and phylogenetic corrections are not considered. Results of the ANCOVA of the different components of demographic resilience against the two axis of human activities (PC1 and PC2; see Fig.1) and mobility. Species are classified as high mobility (range > 100 km) vs. low mobility (< 100 km). Bolded P-values are statistically significant at P < 0.05.

	Df	Sum of squares	F-value	P-value
**Resistance**				
PC1	1.000	3.839	6.892	**0.010**
PC2	1.000	6.753	12.123	**<0.001**
Mobility	1.000	4.040	7.252	**0.008**
**Speed of recovery**				
PC1	1.000	0.690	0.209	0.648
PC2	1.000	3.390	1.030	0.312
Mobility	1.000	31.580	9.589	**0.002**
**Compensation**				
PC1	1.000	0.002	0.004	0.949
PC2	1.000	1.920	4.728	**0.032**
Mobility	1.000	21.502	52.940	**<0.001**

Table S6. Summary of results of the residual method on demographic traits of animals that have a high mobility (> 100 Km range) *vs.* low mobility (< 100 Km range) with correction for matrix dimension. The human presence axis and agricultural land use axis refer to the two main dimensions of human activities from the Human Footprint database, as detailed in Figure 1. Results are provided according to the following format: for each axis of the human pressure, the estimates of the effect size, the standard error, the Z-score and the associated p-value bolded when below 0.05.

"pglmm" for low mobility and high mobility animals								
	Human presence				Agricultural land use			
Trait	*Estimate*	*S.E*	*Z-score*	*P*	*Estimate*	*S.E*	*Z-score*	*P*
**Vital rates**								
Survival	0.038	0.019	2.018	**0.044**	0.077	0.037	2.075	**0.038**
	0.013	0.010	1.340	0.180	0.028	0.019	1.489	0.137
Growth	-0.013	0.016	-0.797	0.426	-0.021	0.031	-0.677	0.498
	-	-	-	-	-	-	-	-
Sexual reproduction	-0.188	0.073	-2.591	**0.010**	-0.276	0.143	-1.927	0.054
	-	-	-	-	-	-	-	-
**Life history traits**								
Net reproductive rate (R_0)	0.012	0.106	0.116	0.907	0.076	0.201	0.378	0.705
	0.034	0.047	0.724	0.469	0.040	0.095	0.420	0.674
Degree of iteroparity (S)	-0.002	0.018	-0.132	0.895	0.001	0.034	0.022	0.982
	-0.004	0.030	-0.124	0.902	-0.044	0.058	-0.758	0.448
Generation time (T)	0.082	0.045	1.826	0.068	0.149	0.089	1.667	0.096
	-0.016	0.029	-0.559	0.576	-0.031	0.055	-0.565	0.572
Age at maturity (L_α)	0.014	0.021	0.658	0.510	0.036	0.043	0.830	0.407
	-0.035	0.024	-1.458	0.145	-0.053	0.046	-1.152	0.249
Rate of actuarial senescence (H)	-0.003	0.004	-0.729	0.466	-0.010	0.008	-1.251	0.211
	-0.008	0.008	-0.991	0.322	-0.020	0.016	-1.242	0.214
**Transient dynamics**								
Damping ratio (ρ)	0.150	0.175	0.856	0.392	0.151	0.333	0.453	0.651
	-0.171	0.135	-1.272	**0.203**	-0.618	0.255	-2.425	**0.015**

**“pglmm” for low mobility and high mobility animals**								
	**Human presence**				**Agricultural land use**			
Trait	*Estimate*	*S.E*	*Z-score*	*P*	*Estimate*	*S.E*	*Z-score*	*P*
Reactivity ($\bar{\rho}_1$)	0.044	0.016	2.783	**0.005**	0.062	0.031	1.986	**0.047**
	0.012	0.010	1.218	0.223	0.027	0.019	1.393	0.164
First step attenuation ($\underline{\rho}_1$)	0.021	0.017	1.239	0.215	0.051	0.034	1.499	0.134
	0.011	0.015	0.690	0.490	0.023	0.029	0.789	0.430
Maximum amplification ($\bar{\rho}_{max}$)	0.045	0.015	2.915	**0.004**	0.062	0.030	2.076	**0.038**
	-	-	-	-	-	-	-	-

Table S7. Summary of the results of the trait analysis for plants (light blue) and animals (light red) using the spatially and phylogenetically corrected model with corrections for matrix dimension and selection of studies that overlap with the Human Footprint Database versions of 1993 and 2009. The human presence axis and agricultural land use axis refer to the two main dimensions of human activities from the Human Footprint database, as detailed in Figure 1. For each axis of the human pressure, the tables shows the estimates of the effect size, the standard error (S.E.), the Z-score, and the associated P-value bolded when below 0.05. Note that none of the examined animal populations include shrinkage or clonal reproduction (thus "-"). Blue background: trait that became significant under the subset data, but was not significant with the full data. Orange: trait that became not significant under the subset data, but was significant with the full data.

**pglmm method for phylogenetically and spatially corrected model for plants and animals**								
	**Human presence axis (PC1)**				**Agricultural land use axis (PC2)**			
Trait	*Estimate*	*S.E.*	*Z-score*	*P*	*Estimate*	*S.E.*	*Z-score*	*P*
**Life history traits**								
Net reproductive rate (R_0)	0.144	0.069	2.086	**0.037**	0.201	0.139	1.450	0.147
	-0.099	0.093	-1.065	0.287	-0.126	0.155	-0.812	0.417
Degree of iteroparity (S)	-0.013	0.033	-0.400	0.689	-0.021	0.066	-0.318	0.750
	-0.004	0.020	-0.223	0.824	-0.029	0.035	-0.831	0.406
Generation time (T)	0.015	0.014	1.034	0.301	0.017	0.029	0.591	0.554
	-0.021	0.101	-0.212	0.832	-0.078	0.175	-0.446	0.656
Age at maturity (L_α)	-0.026	0.053	-0.486	0.627	-0.044	0.105	-0.418	0.676
	0.058	0.124	0.467	0.640	0.059	0.175	0.340	0.734
Rate of actuarial senescence (H)	-0.007	0.009	-0.760	0.447	0.006	0.019	0.330	0.741
	-0.001	0.005	-0.260	0.795	-0.003	0.009	-0.351	0.725
**Transient dynamics**								
Damping ratio (ρ)	0.012	0.036	0.333	0.739	0.015	0.073	0.212	0.832
	-0.005	0.013	-0.403	0.687	-0.009	0.026	-0.360	0.719
Reactivity ($\bar{\rho}_1$)	-0.027	0.030	-0.899	0.369	-0.036	0.064	-0.569	0.569
	0.003	0.026	0.105	0.917	0.008	0.044	0.171	0.864
First step attenuation ($\underline{\rho}_1$)	0.005	0.012	0.412	0.681	0.022	0.024	0.891	0.373
	0.000	0.004	-0.102	0.919	0.000	0.007	-0.065	0.948
Maximum amplification($\bar{\rho}_{max}$)	0.004	0.012	0.366	0.714	0.020	0.024	0.826	0.409
	-0.857	7.632	-0.112	0.911	-0.366	10.427	-0.035	0.972

Table S8. The demographic correlates of human activities occur mostly in animals' demographic processes and life history traits when not correcting for matrix dimension. Summary of the results of the trait analysis for plants (light blue) and animals (light red) using the spatially and phylogenetically corrected model without corrections for matrix dimension. The human presence axis and agricultural land use axis refer to the two main dimensions of human activities from the Human Footprint database, as detailed in Figure 1. Results are provided according to the following format: for each axis of the human pressure, the estimates of the effect size, the standard error, the Z-score and the associated p-value bolded when below 0.05. Note that none of the examined animal populations include shrinkage or clonal reproduction (thus "-").

**"pglmm" method for phylogenetically and spatially corrected model for plants and animals**								
	**Human presence**				**Agricultural land use**			
Trait	*Estimate*	*S.E.*	*Z-score*	*P*	*Estimate*	*S.E.*	*Z-score*	*P*
**Vital rates**								
Survival	0.010	0.005	1.853	0.064	0.011	0.009	1.333	0.182
	0.028	0.008	3.689	**<0.001**	0.048	0.015	3.192	**0.001**
Growth	-0.006	0.005	-1.032	0.302	-0.012	0.009	-1.393	0.164
	0.019	0.008	2.371	**<0.001**	0.033	0.015	2.164	**0.030**
Shrinkage	-0.005	0.004	-1.166	0.244	-0.006	0.006	-0.873	0.383
	-	-	-	-	-	-	-	-
Sexual reproduction	-0.220	0.057	-3.887	**<0.001**	-0.290	0.089	-3.258	**0.001**
	-0.045	0.079	-0.569	0.570	-0.143	0.158	-0.902	0.367
Clonal reproduction	-0.001	0.001	-0.521	0.602	-0.001	0.002	-0.339	0.735
	-	-	-	-	-	-	-	-
**Life history traits**								
Net reproductive rate (R_0)	-0.059	0.072	-0.817	0.414	-0.164	0.116	-1.418	0.156
	0.098	0.093	1.058	0.290	0.146	0.183	0.795	0.427
Degree of iteroparity (S)	-0.006	0.020	-0.301	0.764	-0.013	0.033	-0.400	0.689
	0.084	0.039	2.159	**0.031**	0.215	0.075	2.874	**0.004**
Generation time (T)	0.024	0.032	0.742	0.458	0.055	0.053	1.028	0.304
	0.010	0.022	0.462	0.644	0.003	0.044	0.070	0.944
Age at maturity (L_α)	-0.001	0.014	-0.107	0.915	-0.006	0.021	-0.291	0.771
	-0.023	0.016	-1.467	0.142	-0.061	0.030	-2.069	**0.039**
Rate of actuarial	0.007	0.009	0.731	0.465	0.009	0.015	0.595	0.552
	0.020	0.013	1.512	0.131	0.064	0.026	2.473	**0.013**

"pglmm" method for phylogenetically and spatially corrected model for plants and animals								
	**Human presence**				**Agricultural land use**			
Trait	*Estimate*	*S.E.*	*Z-score*	*P*	*Estimate*	*S.E.*	*Z-score*	*P*
senescence (H)								
**Transient dynamics**								
Damping ratio (ρ)	0.005	0.004	1.106	0.269	0.002	0.007	0.288	0.774
	3.362	10.780	0.312	0.755	-7,698	19,155	-0.402	0.688
Reactivity ($\bar{\rho}_1$)	0.016	0.006	2.483	**0.013**	0.019	0.010	1.870	0.061
	0.018	0.008	2.179	**0.029**	0.017	0.016	1.121	0.262
First step attenuation ($\underline{\rho}_1$)	0.012	0.005	2.251	**0.024**	0.011	0.009	1.322	0.186
	0.012	0.010	1.228	0.219	0.026	0.019	1.344	0.179
Maximum amplification ($\bar{\rho}_{max}$)	-0.107	0.031	-3.459	**<0.001**	-0.129	0.049	-2.649	**0.008**
	0.007	0.008	0.885	0.376	-0.016	0.016	-0.992	0.321

Table S9. List of the demographic traits used in our analysis to understand the underlying factors that may underpin relationships between metrics of demographic resilience and human activities. $\hat{A}$ is the standardized population model obtained by dividing ***A***, the matrix population model, by the dominant eigenvalue λ_1; l_x is the age-specific survivorship schedule; m_x is the age-specific fertility schedule, λ_x is the xth eigenvalue of ***A***.

Trait ID	Trait name	Calculation	Description
**Life history traits**			
R_0	Net reproductive rate	$R_0 = \int_0^\infty l_x m_x dx$	Average number of offspring produced by a female in a population in her lifetime
S	Degree of iteroparity	$S = -e^{-loglog\,(\lambda_1)}\, l_x m_x log(e^{-logl}$	Describes the width of reproductive window, ranging between strictly semelparous (S=0) and highly iteroparous (S>>0)
T	Generation time	$T = \frac{log(R_0)}{log(\lambda_1)}$	Mean age of reproduction in the population
L_α	Age at first reproduction	L_α	Mean age of an individual in the population at the first reproduction
H	Keyfitz' entropy	$H = \frac{-log(l_x) l_x}{\sum \quad l_x}$	Shape of the age-specific survivorship curve l_x as quantified by Keyfitz' entropy (H), corresponding to populations whose mortality hazards increase (H<1), stay constant (H=1) or decrease with age (H>1), respectively
P_{rep}	Probability of reproduction	p_{rep}	Probability for an average individual in the population to reproduce
**Transient dynamics metrics**			

Trait ID	Trait name	Calculation	Description
ρ	Damping ratio	$\rho = \frac{\lambda_1}{\lvert\lambda_2\rvert}$	Speed with which a population returns to a stationary condition after a disturbance (Caswell 2000). A damping ratio of 1 describes a system that returns to stationary conditions effectively immediately. Greater values correspond to an overdamped system which is a system which return to its stationary state slowly without oscillations.
$(\bar{\rho}_1)$	Reactivity	$\bar{\rho}_1 = \lVert\hat{A}\rVert_1$	Maximum population growth rate in a single timestep following a disturbance, relative to the stable growth rate λ_1 (Neubert & Caswell 1997; Stott *et al.* 2011)
$\underline{\rho_1}$	First step attenuation	$\underline{\rho_1} = minCS(\hat{A})$	Minimum population growth in a single timestep following a disturbance, relative to stable growth rate λ_1 (Neubert & Caswell 1997; Stott *et al.* 2011)

Table S10. Representativity of the different kingdoms in our data. For each kingdom, we provide the number of species (# species) in our data, an approximation of the total estimated number of species in each kingdom (# species in Kingdom) and the corresponding taxonomic coverage in each Kingdom (% coverage) of our study.

Kingdom	# species	# species in Kingdom	% coverage
Animalia	47	1,900.000 (Pimm *et al.* 2014)	0.002%
Plantae	300	350,000 (Vellend *et al.* 2017)	0.080%
Fungi	1	44,000 (Mora *et al.* 2011)	0.002%
Algae (Chromista here)	1	18,000 (Mora *et al.* 2011)	0.006%

Table S11. Richness and dispersion of the resilience kernel increase when the agricultural land use intensifies. Richness, dispersion, and evenness values of the kernels for different level of agricultural land use (PC2) ([<-7, -3.5[on the PC2 axis corresponds to low agricultural land use in Figure 1, while [0.5, >4] corresponds to more intensive agricultural land use) without rarefaction analyses. These results, where the length of each segment is equal, resulted in different number of populations in each kernel.

	**[<-7, -3.5[**	**[-3.5, 0.5[**	**[0.5, >4]**
**Richness**	38.094	37.032	77.292
**Dispersion**	2.726	2.546	3.775
**Evenness**	0.060	0.005	0.006

Table S12. Plant population in areas with a high human presence are on average more resistant. Results of the linear regression of the different demographic resilience components along the axis of human activities (Figure 1). This table includes analyses for both plants and animals. Results are shown in the following format: estimation of the effect (t-test, degree of freedom, P-value). Bolded P-values are statistically significant at P < 0.05.

Results of linear regression for plants and animals		
**Component of demographic resilience**	**Human presence axis**	**Agricultural land use axis**
Resistance	-0.033 (t_{788} = -1.891, *P* = 0.059)<br>0.045 (t_{107} = 1.589, *P* = 0.115)	-0.032 ($t788$ = -1.043, *P* = 0.297)<br>0.208 (t_{107} = 3.386, ***P* < 0.001**)
Speed of recovery	0.073 (t_{788} = 7.273, ***P* < 0.001**)<br>0.043 (t_{107} = 0.611, *P* = 0.543)	0.128 (t_{788} = 7.131, ***P* < 0.001**)<br>0.148 (t_{107} = 0.977, *P* = 0.331)
Compensation	0.002 (t_{788} = 0.057, *P* = 0.954)<br>-0.045 (t_{107} = -1.561, *P* = 0.121)	0.091 (t_{788} = 1.690, *P* = 0.091)<br>-0.111 (t_{107} = -1.789, *P* = 0.076)

SI References

Amano, T., González-Varo, J.P. & Sutherland, W.J. (2016). Languages Are Still a Major Barrier to Global Science. *PLOS Biol.*, 14, e2000933.

Blomberg, S.P., Garland, T. & Ives, A.R. (2003). Testing for phylogenetic signal in comparative data: Behavioural traits are more labile. *Evolution*, 57, 717–745.

Caswell, H. (2000). *Matrix population models*. Sunderland MA, USA Sinauer.

Chen, W., Hribar, P. & Melessa, S. (2018). Incorrect Inferences When Using Residuals as Dependent Variables. *J. Account. Res.*, 56, 751–796.

Felsenstein, J. (1985). Phylogenies and the Comparative Method. *Am. Nat.*, 125, 1–15.

Freckleton, R.P. (2002). On the misuse of residuals in ecology: regression of residuals vs. multiple regression. *J. Anim. Ecol.*, 71, 542–545.

Gittleman, J.L. & Kot, M. (1990). Adaptation: Statistics and a Null Model for Estimating Phylogenetic Effects. *Syst. Biol.*, 39, 227–241.

Harvey, P. & Pagel, M. (1991). *The comparative method in evolutionary biology*. Oxford : Oxford university press. Oxford : Oxford university press.

Jin, Y. & Qian, H. (2019). V.PhyloMaker: an R package that can generate very large phylogenies for vascular plants. *Ecography*, 42, 1353–1359.

Kumar, S., Stecher, G., Suleski, M. & Hedges, S.B. (2017). TimeTree: A Resource for Timelines, Timetrees, and Divergence Times. *Mol. Biol. Evol.*, 34, 1812–1819.

Li, D., Dinnage, R., Nell, L.A., Helmus, M.R. & Ives, A.R. (2020). phyr: An r package for phylogenetic species-distribution modelling in ecological communities. *Methods Ecol. Evol.*, 11, 1455–1463.

merrienthomas. (2026). merrienthomas/Online-appendix-Merrien-et-al-Ecology-papper: Online-appendix-Merrien-et-al-Ecology-paper-v2.0 (Version Merrien_et_al_ecology_online_appendix). Zenodo. https://doi.org/10.5281/zenodo.22649459

Mora, C., Tittensor, D.P., Adl, S., Simpson, A.G.B. & Worm, B. (2011). How Many Species Are There on Earth and in the Ocean? *PLOS Biol.*, 9, e1001127.

Moran, P.A.P. (1950). Notes on Continuous Stochastic Phenomena. *Biometrika*, 37, 17–23.

Neubert, M.G. & Caswell, H. (1997). Alternatives to Resilience for Measuring the Responses of Ecological Systems to Perturbations. *Ecology*, 78, 653–665.

OpenTreeofLife, Cranston, K.A., Redelings, B., Reyes, L.L.S., Allman, J., McTavish, E.J., *et al.* (2019). Open Tree of Life Taxonomy.

OpenTreeOfLife, Redelings, B., Reyes, L.L.S., Cranston, K.A., Allman, J., Holder, M.T., *et al.* (2019). Open Tree of Life Synthetic Tree.

Pagel, M. (1999). The Maximum Likelihood Approach to Reconstructing Ancestral Character States of Discrete Characters on Phylogenies. *Syst. Biol.*, 48, 612–622.

Pimm, S.L., Jenkins, C.N., Abell, R., Brooks, T.M., Gittleman, J.L., Joppa, L.N., *et al.* (2014). The biodiversity of species and their rates of extinction, distribution, and protection. *Science*, 344, 1246752.

Stott, I., Townley, S. & Hodgson, D.J. (2011). A framework for studying transient dynamics of population projection matrix models: A synthesis of transient demography. *Ecol. Lett.*, 14, 959–970.
Team, R.C. (2021). *R: A Language and Environment for Statistical Computing*. R Foundation for Statistical Computing.
Vellend, M., Baeten, L., Becker-Scarpitta, A., Boucher-Lalonde, V., McCune, J.L., Messier, J., *et al.* (2017). Plant Biodiversity Change Across Scales During the Anthropocene. *Annu. Rev. Plant Biol.*, 68, 563–586.
Venter, O., Sanderson, E.W., Magrach, A., Allan, J.R., Beher, J., Jones, K.R., *et al.* (2016). Global terrestrial Human Footprint maps for 1993 and 2009. *Sci. Data*, 3, 160067.
